# Identification and Cytotoxic Activities of Two New Trichothecenes and a New Cuparane-Type Sesquiterpenoid from the Cultures of the Mushroom *Engleromyces goetzii*

**DOI:** 10.1007/s13659-014-0051-1

**Published:** 2015-01-30

**Authors:** Yang Wang, Ling Zhang, Gen-Tao Li, Zheng-Hui Li, Ze-Jun Dong, Yan Li, Ji-Kai Liu

**Affiliations:** 1 State Key Laboratory of Phytochemistry and Plant Resources in West China, Kunming Institute of Botany, Chinese Academy of Sciences, Kunming, 650201 China; 2 University of Chinese Academy of Sciences, Beijing, 100049 China

**Keywords:** *Engleromyces goetzii*, Trichothecene, Engleromycone, Cuparane, Infuscol, Mycotoxin

## Abstract

**Abstract:**

*Engleromyces goetzii* is a traditional medicinal mushroom that is widely used to treat infection, inflammation and cancer in Tibet, Sichuan and Yunnan provinces of China. Two new trichothecenes, engleromycones A and B (**1** and **2**), one new cuparane-type sesquiterpenoid named infuscol F (**11**), eight known trichothecene analogs, sambucinol (**3**), 3-deoxysambucinol (**4**), trichothecolone (**5**), trichodermol (**6**), 8-deoxytrichothecin (**7**), trichothecin (**8**), trichothecinol B (**9**) and trichothecinol A (**10**), and one known cyclopentanoid sesquiterpene cyclonerodiol (**12**) were isolated from the cultures of *E. goetzii*. The new compounds were elucidated through spectroscopic analyses. The anticancer effects of trichothecenes **1–10** were examined in the HL-60, SMMC-7721, A549, MCF-7, and SW-480 human cancer cell lines using an MTT assay. Trichothecinol A (**10**) significantly inhibited the growth of MCF-7 cells, with an IC_50_ value of 0.006 µM, which was comparable to the cytotoxic activity of the positive control, paclitaxel, indicating that trichothecinol A (**10**) represents a potential anticancer agent.

**Graphical Abstract:**

**Electronic supplementary material:**

The online version of this article (doi:10.1007/s13659-014-0051-1) contains supplementary material, which is available to authorized users.

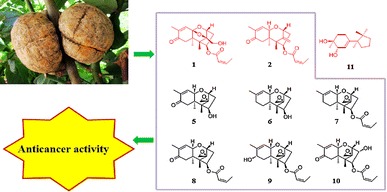

## Introduction

Mushrooms have been valued worldwide as edible and medical provisions for thousands of years. Several mushrooms, such as *Ganoderma lucidum, Hericium erinaceus* and *Antrodia cinnamomea*, have historically been used in Asia for medicinal purposes [1, 2]. *Engleromyces goetzii*, which belongs to the family Hypocreaceae, is a traditional medicinal mushroom that is primarily distributed in Tibet, Sichuan and Yunnan provinces. This fungus typically grows on bamboo in high mountains, and it ripens during the rainy season from July to August. Local residents can purchase this fungus at the market and boil the fruiting bodies in water to treat infection, inflammation and cancer [3–5]. The positive curative effects of *E. goetzii* have garnered interest regarding the chemical constituents of this mushroom. Previous investigations on the fruiting bodies of this fungus have led to the isolation of neoengleromycin, cytochalasin D and 19,20-epoxycytochalasin D [4, 6, 7]. However, the chemical constituents of the cultures of this fungus have not been previously reported. Therefore, we conducted an initial investigation on *E. goetzii* cultures, which resulted in the isolation of two trichothecenes (**4** and **8**). In previous studies, trichothecin (**8**) exhibited promising anticancer activity [8].

Trichothecenes are a series of mycotoxins produced by several fungi, with genera including *Fusarium*, *Mycothecium*, *Trichoderma*, *Trichothecium*, *Stachybotrys* and *Cephalosporium* [9, 10]. These fungi typically infest maize, oats, barley and wheat, producing trichothecenes [9]. Currently, more than 150 trichothecenes have been reported [10]. Trichothecenes are an important source of contamination in food and feed [11, 12]. When contaminated food and feed are ingested, trichothecenes initiate a wide range of acute and chronic symptoms, including cardiovascular lesions, hypotension, anemia and lymphoid necrosis [13, 14]. The effects of trichothecenes on eukaryotic cells include the inhibition of protein, DNA and RNA synthesis, inhibition of mitochondrial functions and cell division, and membrane effects [14, 16]. Moreover, trichothecenes exhibit multiple biological activities, such as antibiotic, antibacterial, antiviral and antitumor activities [14, 15].

**Fig. 1:**
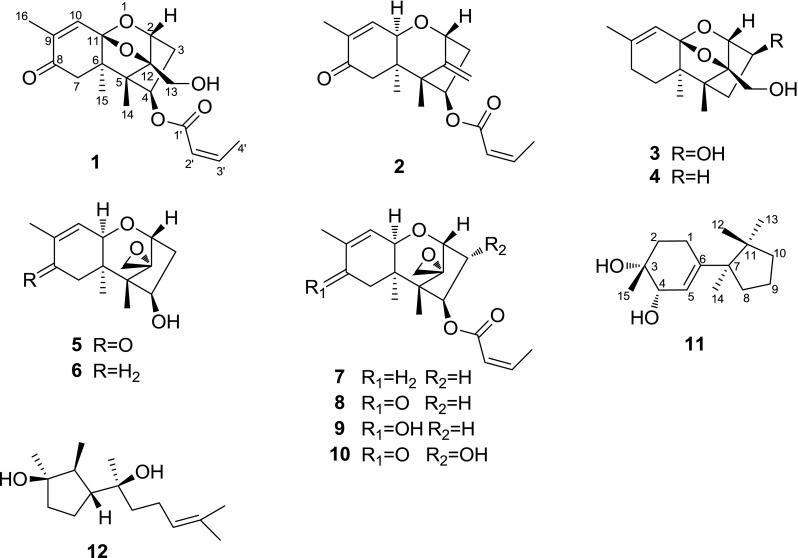
Structures of compounds **1**–**12**

To identify additional novel and potentially bioactive secondary metabolites, the chemical constituents of *E. goetzii* cultures were investigated by altering the culture conditions of the fungus and increasing the fermentation scale. This investigation led to the isolation and identification of two new trichothecenes named engleromycones A and B (**1** and **2**), one new cuparane-type sesquiterpenoid named infuscol F (**11**) (Fig. 1), eight known trichothecene analogs, sambucinol (**3**), 3-deoxysambucinol (**4**), trichothecolone (**5**), trichodermol (**6**), 8-deoxytrichothecin (**7**), trichothecin (**8**), trichothecinol B (**9**) and trichothecinol A (**10**), and one known cyclopentanoid sesquiterpene cyclonerodiol (**12**). The structures of the compounds were elucidated based on spectroscopic analyses. Cytotoxicity assays against the HL-60, SMMC-7721, A549, MCF-7 and SW-480 human cancer cell lines indicated that compounds **1**, **2**, and **5**–**10** significantly reduced the viabilities of these cells. In particular, trichothecinol A (**10**) exhibited the strongest inhibitory effect on the growth of MCF-7 cells, with an IC_50_ value of 0.006 µM, which was comparable to the cytotoxic activity of the positive control, paclitaxel. An analysis of the structure-cytotoxicity relationships of trichothecenes **1**–**10** suggested that the 12,13-epoxide ring, the hydroxyl group at C-3 and the –OCOCH=CHCH_3_ substituent at C-4 clearly enhance the cytotoxic activity. This study describes the isolation and structure elucidation of the isolates and the cytotoxic activities and structure–cytotoxicity relationships of the trichothecenes.

## Results and Discussion

### Structure Elucidation

**Fig. 2:**
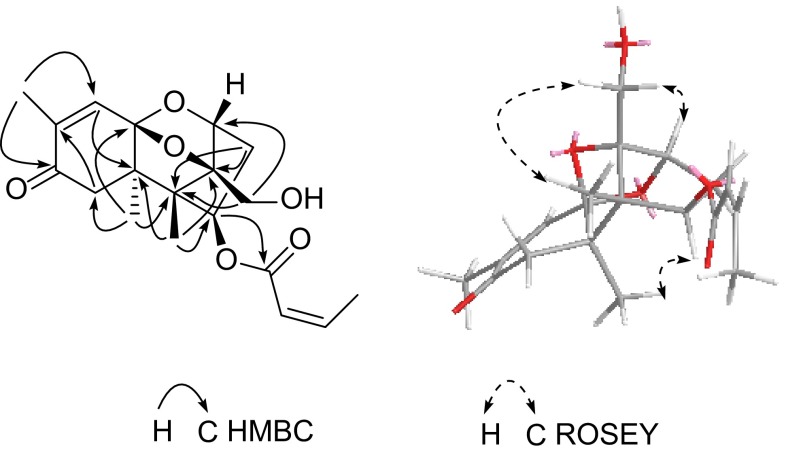
Selected 2D NMR correlations of **1**

Compound **1** is a colorless oil and exhibited an [M + Na]^+^ ion at *m*/*z* 371.1472 in positive HR ESI MS analysis, corresponding to the molecular formula C_19_H_24_O_6_ with 8° of unsaturation. The ^1^H NMR spectrum of **1** exhibited four methyl groups at *δ*_H_ 0.91 (3H, s), 1.08 (3H, s), 1.84 (3H, s) and 2.14 (3H, dd, *J* = 7.2, 1.8 Hz) and three olefinic protons at *δ*_H_ 5.74 (1H, dd, *J* = 11.4, 1.8 Hz) and 6.40 (2H, overlapped). A careful analysis of the ^1^H NMR signals at *δ*_H_ 5.74 (1H, dd, *J* = 11.4, 1.8 Hz), 6.40 (1H, overlapped), and 2.14 (3H, dd, *J* = 7.2, 1.8 Hz) and the ^13^C NMR (DEPT) signals at *δ*_C_ 165.2 (s), 119.7 (d), 146.9 (d), and 15.4 (q) revealed a –OCOCH=CHCH_3_ substituent group. The remaining NMR signals indicated three methyl groups, three methylenes, three methines and six quaternary carbons, suggesting that **1** was a sesquiterpene. The ^1^H and ^13^C NMR spectral data of **1** were similar to those of loukacinal A [17], which confirmed that **1** is a trichothecene. However, there are certain differences, as follows: the resonance of H-4 at *δ*_H_ 4.30 in loukacinal A is downshifted to *δ*_H_ 5.32 in **1**, suggesting that the hydroxyl at C-4 is esterified. The methane at *δ*_H_ 5.32 (1H, dd, *J* = 7.7, 2.0 Hz) exhibited an HMBC correlation with the carbons at *δ*_C_ 165.2, indicating that the –OCOCH=CHCH_3_ substituent group is attached to C-4 (Fig. 2). In the ROSEY spectrum, the observed cross peaks of H-2/H-13, H-13/CH_3_-14, and H-4/CH_3_-15 suggested that H-2, H-13 and CH_3_-14 lie on the same side, whereas H-4 and CH_3_-15 lie on the opposite side (Fig. 2). Therefore, **1** was determined as engleromycone A.

**Fig. 3:**
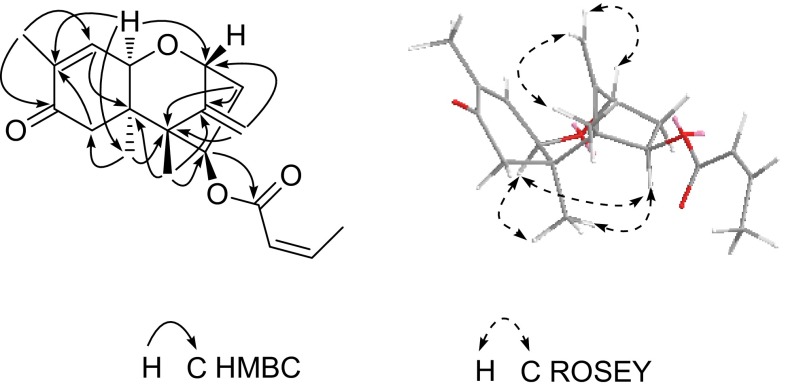
Selected 2D NMR correlations of **2**

Compound **2** was obtained as a colorless oil with a molecular formula of C_19_H_24_O_4_ based on the HR ESI MS ion at *m*/*z* 339.1563 ([M + Na]^+^), requiring 8° of unsaturation. The 1D NMR spectroscopic data (Table 1) suggested that the backbone of **2** was identical to that of **1**. One of the differences between these compounds was identified as the loss of a quaternary carbon and the appearance of a methine. In HMBC analysis (Fig. 3), the proton signal at *δ*_H_ 3.95 correlated with the carbons at *δ*_C_ 18.4, 79.3 and 138.0, suggesting that the oxygen linkage between C-11 and C-12 disappeared, and a methine was present at C-11. Moreover, the proton signals at *δ*_H_ 5.22 and 4.81 correlated with the carbons at *δ*_C_ 79.3 and 51.7 (Fig. 3), indicating that a double bond was formed between C-12 and C-13. In the ROSEY spectrum, the observed cross peaks of H-4/H-11, H-11/CH_3_-15, CH_3_-15/H-4, H-13a/CH_3_-14 and H-2/H-13b suggested that H-4, H-11 and CH_3_-15 lie on the same side, whereas H-2, H-13 and CH_3_-14 lie on the opposite side (Fig. 3). Therefore, compound **2** was determined as engleromycone B.

**Table 1:** ^1^H NMR (500 MHz) and ^13^C NMR (125 MHz) data for compounds **1** and **2** in CDCl_3_

	**1**		**2**	
	*δ*_C_ type	*δ*_H_ (*J* in Hz)	*δ*_C_ type	*δ*_H_ (*J* in Hz)
2	79.9, d	4.42, d (5.1)	79.3, d	4.54, d (5.2)
3	38.1, t	2.63, dd (16.8, 7.7)	37.9, t	2.64, dd (15.6, 7.6)
		1.89, ddd (16.8, 5.1, 2.0)		1.83, ddd (15.6, 5.2, 3.5)
4	76.2, d	5.32, dd (7.7, 2.0)	73.4, d	5.54, dd (7.6, 3.5)
5	53.6, s		51.7, s	
6	54.8, s		43.6, s	
7	47.7, t	2.74, d (15.0)	41.6, t	2.79, d (16.0)
		2.28, d (15.0)		2.20, d (16.0)
8	198.5, s		199.5, s	
9	141.4, s		138.0, s	
10	133.5, d	6.40, overlapped	137.3, d	6.47, dd (5.7, 1.4)
11	105.9, s		69.6, d	3.95, d (5.7)
12	96.7, s		151.5, s	
13	58.6, t	4.10, d (12.5)	107.0, t	5.22, s, H-b
		3.90, dd (12.5, 7.5)		4.81, s, H-a
14	10.9, q	0.91, s	10.0, q	0.93, s
15	17.4, q	1.08, s	18.4, q	1.05, s
16	15.4, q	1.84, s	15.5, q	1.78, s
1′	165.2, s		165.9, s	
2′	119.7, d	5.74, dd (11.4, 1.8)	120.2, d	5.76, dd (11.4, 1.8)
3′	146.9, d	6.40, overlapped	146.1, d	6.36, dq (11.4, 7.4)
4′	15.4, q	2.14, dd (7.2, 1.8)	15.5, q	2.13, dd (7.4, 1.8)
13-OH		2.01, brs		

**Fig. 4:**
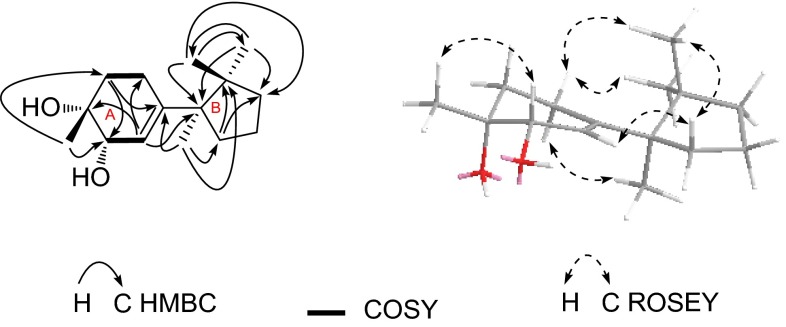
Selected 2D NMR correlations of **11**

Compound **11** possessed the molecular formula C_15_H_26_O_2_ as determined by HR ESI MS analysis (*m*/*z* 261.1825 [M + Na]^+^), which indicated 3° of unsaturation. The ^1^H NMR spectrum indicated four methyl groups at *δ*_H_ 0.79 (3H, s), 0.99 (3H, s), 1.02 (3H, s) and 1.13 (3H, s) and one olefinic proton at *δ*_H_ 5.44 (1H, m). The ^1^H and ^13^C NMR spectra indicated a trisubstituted double bond (*δ*_H_ 5.44, m; *δ*_C_123.8, d; and *δ*_C_ 146.0, s), an oxygen-bearing methine (*δ*_H_ 3.74, m; *δ*_C_ 72.2, d), an oxygen-bearing quaternary carbon (*δ*_C_ 69.2), four tertiary methyls, five methylenes and two quaternary carbons. In HMBC analysis, the proton signal at *δ*_H_ 1.13 correlated with the carbons at *δ*_C_ 33.8 and 72.2, the proton signals at *δ*_H_ 5.44 correlated with the carbons at *δ*_C_ 25.8 and 69.2, and the proton signals at *δ*_H_ 1.73 and 1.48 correlated with the carbons at *δ*_C_ 146.0 and 72.2. In the ^1^H-^1^H COSY spectrum, the signals at *δ*_H_ 1.73 and 1.48 correlated with *δ*_H_ 1.96 and 2.31, and *δ*_H_ 3.74 correlated with *δ*_H_ 5.44, indicating the fragments of –CH_2_–CH_2_– and –CH–CH–. These correlations revealed the presence of structural ring A. Moreover, the HMBC correlations between *δ*_H_ 0.99 and *δ*_C_ 37.4, 44.3; *δ*_H_ 1.02 and *δ*_C_ 51.4, 41.0, and 26.4; *δ*_H_ 2.17, 1.41 and *δ*_C_ 44.3, 41.0; and *δ*_H_ 0.79 and *δ*_C_ 51.4, 41.0, and 25.1 indicated the presence of structural ring B. Furthermore, the proton signals at *δ*_H_ 5.44 exhibited an HMBC correlation with carbon *δ*_C_ 51.4, and *δ*_H_ 0.99 exhibited an HMBC correlation with carbon *δ*_C_ 146.0, suggesting that structural rings A and B were joined through carbons *δ*_C_ 146.0 and 51.4 (Fig. 4). Based on this analysis, the structure of **11** was presumed to be a cuparane-type sesquiterpenoid. The ROSEY spectrum exhibited cross peaks between H-5/H-8*β*, H-8*β*/CH_3_-12, H-1*β*/CH_3_-12, H-1*α*/CH_3_-14and H-4/CH_3_-15 (Fig. 4). A detailed analysis of the chemical shifts and coupling constants led to the determination of 3α,4α-dihydroxy-5-cuparene as infuscol F (**11**).

Based on spectroscopic analyses and comparison with the literature, the known compounds were identified as sambucinol (**3**) [18], 3-deoxysambucinol (**4**) [18], trichothecolone (**5**) [19], trichodermol (**6**) [19], 8-deoxytrichothecin (**7**) [20], trichothecin (**8**) [19], trichothecinol B (**9**) [8], trichothecinol A (**10**) [17], and cyclonerodiol (**12**) [21].

### Effects of Compounds **1**–**10** on the Growth of the HL-60, SMMC-7721, A549, MCF-7 and SW-480 Cell Lines

Compounds **1**–**10** were assayed for their cytotoxicity against five human cancer cell lines (HL-60, SMMC-7721, A549, MCF-7 and SW-480) using the MTT assay in vitro, with cisplatin and taxol as the positive controls. The results are shown in Table 2. Following a 48-h treatment, trichodermol (**6**), 8-deoxytrichothecin (**7**), trichothecin (**8**), trichothecinol B (**9**) and trichothecinol A (**10**) significantly reduced the cell viabilities of these five cell lines, with IC_50_ values lower than 3.5 µM, suggesting that the inhibitory effects of these compounds were more potent than those of the positive control, cisplatin. In particular, trichothecinol A (**10**) exhibited the strongest inhibitory effect on the growth of HL-60, SMMC-7721, A549, MCF-7 and SW-480 cells, with IC_50_ values of 0.022, 0.020, 0.015, 0.006, and 0.011 µM, respectively, which was comparable to that of the positive control, paclitaxel. Engleromycone B (**2**) and trichothecolone (**5**) exhibited clear cytotoxic activity, with IC_50_ values ranging from 2.7 to 21.13 µM. Engleromycone A (**1**) exhibited moderate cytotoxic activity, with IC_50_ values ranging from 14.29 to 20.22 µM. Sambucinol (**3**) and 3-deoxysambucinol (**4**) exhibited weaker cytotoxic activity against these five cancer cell lines, with IC_50_ values greater than 40 µM, and can be regarded as inactive.

**Table 2:** Inhibitory effect of the isolated compounds against different cancer cell lines

Compd.	Cancer cell line IC_50_ (μM)				
	HL-60	SMMC-7721	A549	MCF-7	SW480
**1**	14.29	19.12	19.10	18.04	20.22
**2**	2.70	8.41	2.98	21.13	9.25
**3**	>40	>40	>40	>40	>40
**4**	>40	>40	>40	>40	>40
**5**	4.86	6.92	4.16	16.49	8.52
**6**	1.53	2.14	1.01	3.43	1.87
**7**	0.10	0.15	0.14	0.23	0.15
**8**	0.14	0.22	0.20	0.21	0.18
**9**	1.68	0.45	0.40	0.54	0.35
**10**	0.022	0.020	0.015	0.006	0.011
Cisplatin	1.91	5.81	6.43	13.26	11.99
Taxol	<0.008	<0.008	<0.008	<0.008	<0.008

### Structure-Cytotoxicity Relationships

Trichothecin (**8**) exhibited stronger cytotoxicity than engleromycone A (**1**), suggesting that an epoxide at C-12 and C-13 increases the cytotoxicity. Moreover, trichothecinol A (**10**) exhibited greater potential cytotoxic activity than that of trichothecin (**8**), indicating that the hydroxyl at C-3 increases the cytotoxic activity. Additionally, a comparison between 8-deoxytrichothecin (**7**) and trichodermol (**6**) and between trichothecin (**8**) and trichothecolone (**5**) indicated that compounds with a –OCOCH=CHCH_3_ substituent at C-4 (**7** and **8**) exhibited stronger cytotoxic activities than those with a hydroxyl at C-4 (**5** and **6**). Finally, the differences in the cytotoxic activities among 8-deoxytrichothecin (**7**), trichothecin (**8**) and trichothecinol B (**9**) were small, indicating that alterations in the substituent at C-8 do not effectively improve the cytotoxic activity.

### Selective Cytotoxicities of the Trichothecenes Against Cancer Cells

To investigate the selective cytotoxicities of the trichothecenes, we chose A549 as the cancer cell line and BEAS-2B as the normal cell line. The cytotoxicities of compounds **1**, **2** and **5**–**10** were examined on these two cell lines using the MTT assay. The results indicated that the cytotoxicities of these compounds against the two cell lines were similar (Table 3), suggesting that compounds **1**, **2** and **5**–**10** did not exhibit selective cytotoxicity against the A549 cell line.

**Table 3:** Inhibitory effect of compounds **1**, **2** and **5**–**10** against A-549 and BEAS-2B cell Lines

Compd.	Cancer cell line IC_50_ (μM)	
	A549	BEAS-2B
**1**	18.94	19.42
**2**	5.82	9.87
**5**	5.71	4.18
**6**	1.54	1.06
**7**	0.15	0.11
**8**	0.22	0.27
**9**	0.56	0.45
**10**	0.016	0.025
Cisplatin	9.72	9.53
Paclitaxel	<0.008	<0.98

In summary, three new and nine known sesquiterpenoids were successfully isolated from the cultures of *E. goetzii.* The biological studies indicated that compounds **1**, **2** and **5**–**10** significantly reduced the viabilities of five human cancer cell lines (HL-60, SMMC-7721, A549, MCF-7, and SW-480). In particular, trichothecinol A (**10**) exhibited the strongest inhibitory effect on the growth of MCF-7 cells, with an IC_50_ value of 0.006 µM, which was comparable to the cytotoxic activity of the positive control, paclitaxel. These exciting results suggested that the trichothecene analogs represent promising models for the design of new anticancer agents. Therefore, we further investigated the selective cytotoxicity of compounds **1**, **2** and **5**–**10** against cancer cells and normal cells. Although compounds **1**, **2** and **5**–**10** did not exhibit selective cytotoxicities, additional investigation to improve the selective cytotoxicities of these trichothecenes would promote their use as a series of potent anticancer agents.

## Materials and Methods

### General Experimental Procedures

The optical rotations were measured on a JASCO model 1020 polarimeter (JASCO International Co. Ltd., Tokyo, Japan). The UV spectra were obtained on a Shimadzu double-beam 2401A spectrophotometer (Shimadzu, Kyoto, Japan). The IR spectra were recorded on a Bruker TENSOR 27 FT-IR spectrometer (Bruker, Ettlingen, Germany) with KBr pellets. The 1D and 2D NMR data were acquired on Bruker AM-400, DRX-500 and AV-600 instruments at room temperature (Bruker, Rheinstetten, Germany). The chemical shifts (*δ*) are expressed in ppm with reference to the solvent signals. The mass spectra (MS) were obtained on an API QSTAR time-of-flight mass spectrometer (MDS Sciex, Ontario, Canada) or a VG Autospec-3000 spectrometer (VG, Manchester, England). Silica gel (200–300 mesh, Qingdao Marine Chemical Inc., Qingdao, China), Sephadex LH-20 (Amersham Biosciences, Sweden), and RP-18 gel (40–75 µM, Fuji Silysia Chemical Ltd., Japan) were used for column chromatography. Preparative HPLC (Prep-HPLC) was performed on an Agilent 1100 liquid chromatography system equipped with a ZORBAX SB-C_18_column (9.4 mm × 150 mm). Precoated silica gel GF 254 plates (Qingdao Marine Chemical Inc., Qingdao, China) were used for TLC analysis. The fractions were monitored by TLC analysis, and the spots were visualized under UV light (254 or 365 nm) or by heating silica gel plates sprayed with 10 % H_2_SO_4_ in ethanol.

### Fungal Material and Cultivation Conditions

The fungus *E. goetzii* was collected from bamboo groves of Shangri-La County in Yunnan Province, China, in August 2012 and was identified by Prof. Zhu-Liang Yang (Kunming Institute of Botany, Chinese Academy of Sciences). A voucher specimen was deposited in the Herbarium of the Kunming Institute of Botany of the Chinese Academy of Sciences. The mycelial cultures were derived from the tissue plugs. The culture PDA medium consisted of glucose (5 %), peptone from porcine meat (0.15 %), yeast powder (0.5 %), KH_2_PO_4_ (0.05 %) and MgSO_4_ (0.05 %). The inoculums of *E. goetzii* were prepared in a 15-L fermenter (Biostar, Shanghai Guoqiang, China) for 6 d under the following conditions: culture temperature, 24 °C; initial pH, 6.0; agitation speed, 250 r/min; inoculation volume, 10 % (by volume); and aeration rate, 1.0 volume/culture volume/min. Subsequently, the liquid seed was transferred into a 100-L fermentation tank for cultivation under identical conditions for 20 d to afford an 80-L culture broth.

### Extraction and Isolation

The fermentation broth (80 L) was filtered, and the filtrate was concentrated to 10 L under reduced pressure and then extracted with ethyl acetate (3 × 10 L). The organic layer was evaporated to yield a crude extract (350 g). Subsequently, the extract was subjected to silica gel column chromatography using a petroleum ether/acetone gradient system (100:0 → 0:100 V/V) to afford fractions F_1_–F_7_ based on TLC analyses. F_2_ was purified using Sephadex LH-20 column chromatography (chloroform/methanol = 1:1 V/V) and then subjected to silica gel column chromatography (petroleum ether/acetone = 150:1 V/V) to afford compound **8** (2160 mg). F_3_ was fractionated using Sephadex LH-20 column chromatography (chloroform/methanol = 1:1 V/V) to yield two subfractions (F_3–1_ and F_3–2_). F_3–2_ was subjected to silica gel column chromatography using a petroleum ether/acetone system (100:1 V/V) to yield F_3–2–1_ and F_3–2–2_. F_3–2–2_ was purified by preparative HPLC (acetonitrile/water = 6:4 → 9:1 V/V, 10 mL/min in 20 min) to afford compounds **2** (7.0 mg, retention time (t_*R*_) = 11.2 min) and **7** (28.8 mg, t_*R*_ = 12.3 min). F_4_ was subjected to silica gel column chromatography and eluted by chloroform/acetone (150:1 V/V), followed by Sephadex LH-20 column chromatography (chloroform/methanol = 1:1 V/V) to afford compound **4** (80.0 mg). F_5_ was separated using silica gel column chromatography (chloroform/methanol = 100:1 V/V) to yield two fractions (F_5–1_ and F_5–2_). F_5–1_ was further purified using Sephadex LH-20 column chromatography (chloroform/methanol = 1:1 V/V) to afford compound **12** (40.9 mg). F_5–2_ was separated with silica gel (chloroform/methanol = 150:1 V/V) and Sephadex LH-20 (chloroform/methanol = 1:1 V/V) column chromatography to yield two subfractions (F_5–2–1_ and F_5–2–2_). F_5–2–1_was separated by preparative HPLC (acetonitrile/water = 2:8 → 6:4 V/V, 10 mL/min in 25 min) to afford compounds **1** (27.5 mg, t_*R*_ = 16.5 min), **6** (2.6 mg, t_*R*_ = 6.5 min), **9** (36.3 mg, t_*R*_ = 7.9 min) and **10** (14.3 mg, t_*R*_ = 9.5 min). F_5–2–2_ was purified by silica gel column chromatography (chloroform/methanol = 100:1 V/V) to afford compound **3** (18.2 mg). F_6_ was subjected to silica gel column chromatography (chloroform/methanol = 50:1 V/V), followed by Sephadex LH-20 column chromatography (chloroform/methanol = 1:1 V/V) to afford compound **5** (1426 mg). F_7_ was purified by Sephadex LH-20 (chloroform/methanol = 1:1 V/V) and RP-18 gel (methanol/water = 1:1 V/V) column chromatography to afford compound **11** (0.8 mg).

### Engleromycone A (**1**)

Colorless oil; $\left[ \alpha \right]_{\text{D}}^{22}$ + 1.7 (*c* 0.18, MeOH); UV (MeOH): λ_max_ (log ε) 372 (1.71), 212 (3.96) nm; IR (KBr): ν_max_ 3472, 3457, 1719, 1686, 1645, 1171, 1046 cm^−1^; ^1^H and ^13^C NMR data see Table 1; ESI MS (positive) *m*/*z* 371 (100) [M + Na]^+^; 349 (60) [M + H]^+^; 719 (15) [2M + Na]^+^; HR ESI MS (positive) *m*/*z* 371.1,472 [M + Na]^+^ (calculated for C_19_H_24_O_6_Na, 371.1471).

### Engleromycone B (**2**)

Colorless oil; $\left[ \alpha \right]_{\text{D}}^{22}$ + 7.9 (*c* 0.2, MeOH); UV (MeOH): λ_max_ (log ε) 211 (3.46) nm; IR (KBr): ν_max_ 2984, 2940, 2926, 1713, 1650, 1437, 1415, 1181, 1026 cm^−1^; ^1^H and ^13^C NMR data see Table 1; ESI MS (positive) *m*/*z* 339 (30) [M + Na]^+^; 655 (10) [2M + Na]^+^; HR ESI MS (positive) *m*/*z* 339.1563 [M + Na]^+^ (calculated for C_19_H_24_O_4_Na, 339.1572).

### Infuscol F (**11**)

Colorless oil; $\left[ \alpha \right]_{\text{D}}^{22}$ + 46.3 (*c* 0.08, MeOH); UV (MeOH): λ_max_ (log ε) 203 (3.97) nm; IR (KBr): ν_max_ 3441, 3265, 2957, 2933, 2875, 1640, 1413 cm^−1^; ^1^H and ^13^C NMR data see Table 4; ESI MS (positive) *m*/*z* 261 (100) [M + Na]^+^; 499 (50) [2M + Na]^+^; HR ESI MS (positive) *m*/*z* 261.1825 [M + Na]^+^ (calculated for C_15_H_26_O_2_Na, 261.1830).

**Table 4:** ^1^HNMR (600 MHz) and ^13^C NMR (150 MHz) data for compound **11** in acetone-*d*_6_

	*δ*_C_ type	*δ*_H_ (*J* in Hz)
1	25.8, t	2.31, m, H-α
		1.96, m, H-β
2	33.8, t	1.73, ddd (12.8, 6.9, 5.4)
		1.48, overlapped
3	69.2, s	
4	72.2, d	3.74, m
5	123.8, d	5.44, m
6	146.0, s	
7	51.4, s	
8	37.4 t	2.17, m, H-β
		1.41, m, H-α
9	19.7, t	1.62, overlapped
10	41.0, t	1.62, overlapped
		1.49, overlapped
11	44.3, s	
12	25.1, q	1.02, s
13	26.4, q	0.79, s
14	22.7, q	0.99, s
15	25.4, q	1.13, s

### Cytotoxicity Assays

The human tumor cell lines HL-60 (promyelocytic leukemia), SMMC-7712 (hepatocarcinoma), A549 (lung carcinoma), MCF-7 (breast adenocarcinoma), SW-480 (colon adenocarcinoma) and BEAS-2B normal cell line (human bronchial epithelial cell) were used. All of the cells were cultured in RMPI-1640 or DMEM medium (HyClone, Logan, UT, USA) plus 10 % fetal bovine serum (HyClone) at 37 °C in a humidified atmosphere with 5 % CO_2_. The cytotoxicity assay was performed according to the MTT (3-(4,5-dimethylthiazol-2-yl)-2,5-diphenyltetrazolium bromide) method in 96-well microplates. Briefly, 100 µL of adherent cells was seeded into each well of a 96-well cell culture plate and allowed to adhere for 12 h prior to drug addition, whereas the suspended cells were seeded immediately prior to drug addition, both with an initial density of 1 × 10^5^ cells/mL. Each tumor cell line was exposed to the test compounds at various concentrations in triplicate for 48 h, with cisplatin and paclitaxel as the positive controls. Following incubation, MTT (100 µg) was added to each well, and the incubation continued for 4 h at 37 °C. The cells were lysed with 200 µL of SDS following the removal of 100 µL of the medium. The optical density of the lysate was measured at 595 nm in a 96-well microtiter plate reader (Bio-Rad 680). The IC_50_ value of each compound was calculated using Reed and Muench’s method [22].

## Electronic supplementary material

Below is the link to the electronic supplementary material.

Supplementary material 1 (PDF 1496 kb)
