# Supplementary material for: Identification and Cytotoxic Activities of Two New Trichothecenes and a New Cuparane-Type Sesquiterpenoid from the Cultures of the Mushroom Engleromyces goetzii
Source: Nat Prod Bioprospect. 2015 Jan 30;5(1):47–53. doi: 10.1007/s13659-014-0051-1 (PMC4328002; doi:10.1007/s13659-014-0051-1)
Supplement: Supplementary file 1 — Supplementary material 1 (PDF 1496 kb) [file 13659_2014_51_MOESM1_ESM.pdf]

Supporting Information

Identification and cytotoxic activities of trichothecenes and a cuparane-type sesquiterpenoid from the cultures of the mushroom *Engleromyces goetzii*

Yang Wang,[†,‡] Ling Zhang,[†] Gentao Li,[†] Zhenghui Li,[†] Zejun Dong,[†] Yan Li,[†] and Jikai Liu[*,†]

[†]State Key Laboratory of Phytochemistry and Plant Resources in West China, Kunming Institute of Botany, Chinese Academy of Sciences, Kunming 650201, China

[‡] University of Chinese Academy of Sciences, Beijing 100049, China

Contents:

S1. ^{1}H NMR spectrum (500 MHz, $CDCl_3$) of engleromycone A (**1**).

S2. ^{13}C NMR spectrum (100 MHz, $CDCl_3$) of engleromycone A (**1**)

S3. HSQC spectrum (500 MHz, $CDCl_3$) of engleromycone A (**1**)

S4. HMBC spectrum (500 MHz, $CDCl_3$) of engleromycone A (**1**)

S5. COSY spectrum (500 MHz, $CDCl_3$) of engleromycone A (**1**)

S6. ROSEY spectrum (500 MHz, $CDCl_3$) of engleromycone A (**1**)

S7. HR-ESI-MS spectrum of engleromycone A (**1**)

S8. ^{1}H NMR spectrum (500 MHz, $CDCl_3$) of engleromycone B (**2**)

S9. ^{13}C NMR spectrum (100 MHz, $CDCl_3$) of engleromycone B (**2**)

S10. HSQC spectrum (500 MHz, $CDCl_3$) of engleromycone B (**2**)

S11. HMBC spectrum (500 MHz, $CDCl_3$) of engleromycone B (**2**)

S12. COSY spectrum (500 MHz, $CDCl_3$) of engleromycone B (**2**)

S13. ROSEY spectrum (500 MHz, $CDCl_3$) of engleromycone B (**2**)

S14. HR-ESI-MS spectrum of engleromycone B (**2**)

S15. ^{1}H NMR spectrum (600 MHz, acetone-d_6) of infuscol F (**11**)

S16. ^{13}C NMR spectrum (150 MHz, acetone-d_6) of infuscol F (**11**)

S17. HSQC spectrum (600 MHz, acetone-d_6) of infuscol F (**11**)

S18. HMBC spectrum (600 MHz, acetone-d_6) of infuscol F (**11**)

S19. COSY spectrum (600 MHz, acetone-d_6) of infuscol F (**11**)

S20. ROSEY spectrum (600 MHz, acetone-d_6) of infuscol F (**11**)

S21. HR-ESI-MS spectrum of infuscol F (**11**)

S1. ^{1}H NMR spectrum (500 MHz, $CDCl_3$) of engleromycone A (**1**)

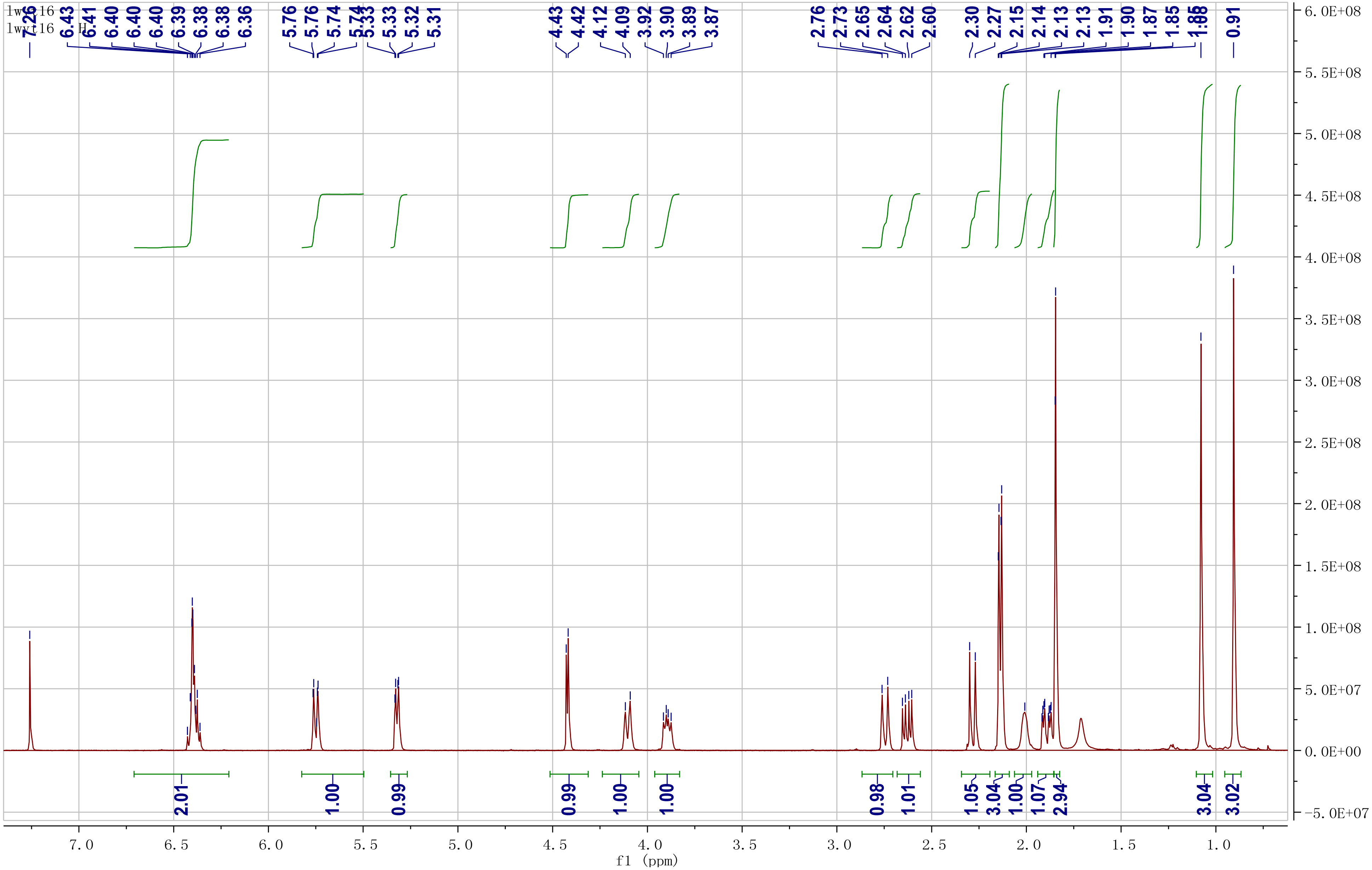

S2. ^{13}C NMR spectrum (100 MHz, $CDCl_3$) of engleromycone A (**1**)

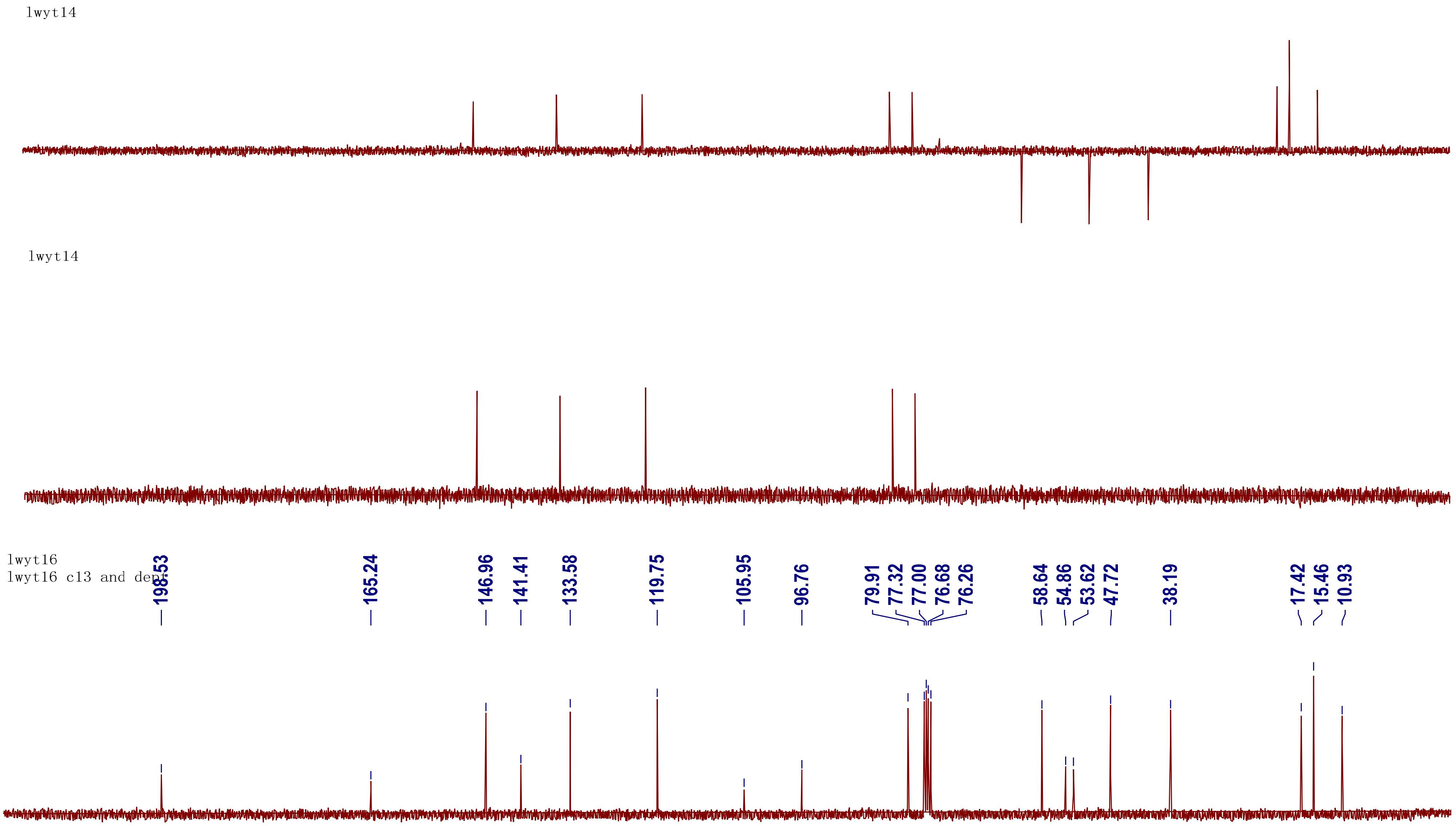

S3. HSQC spectrum (500 MHz, $CDCl_3$) of engleromycone A (**1**)

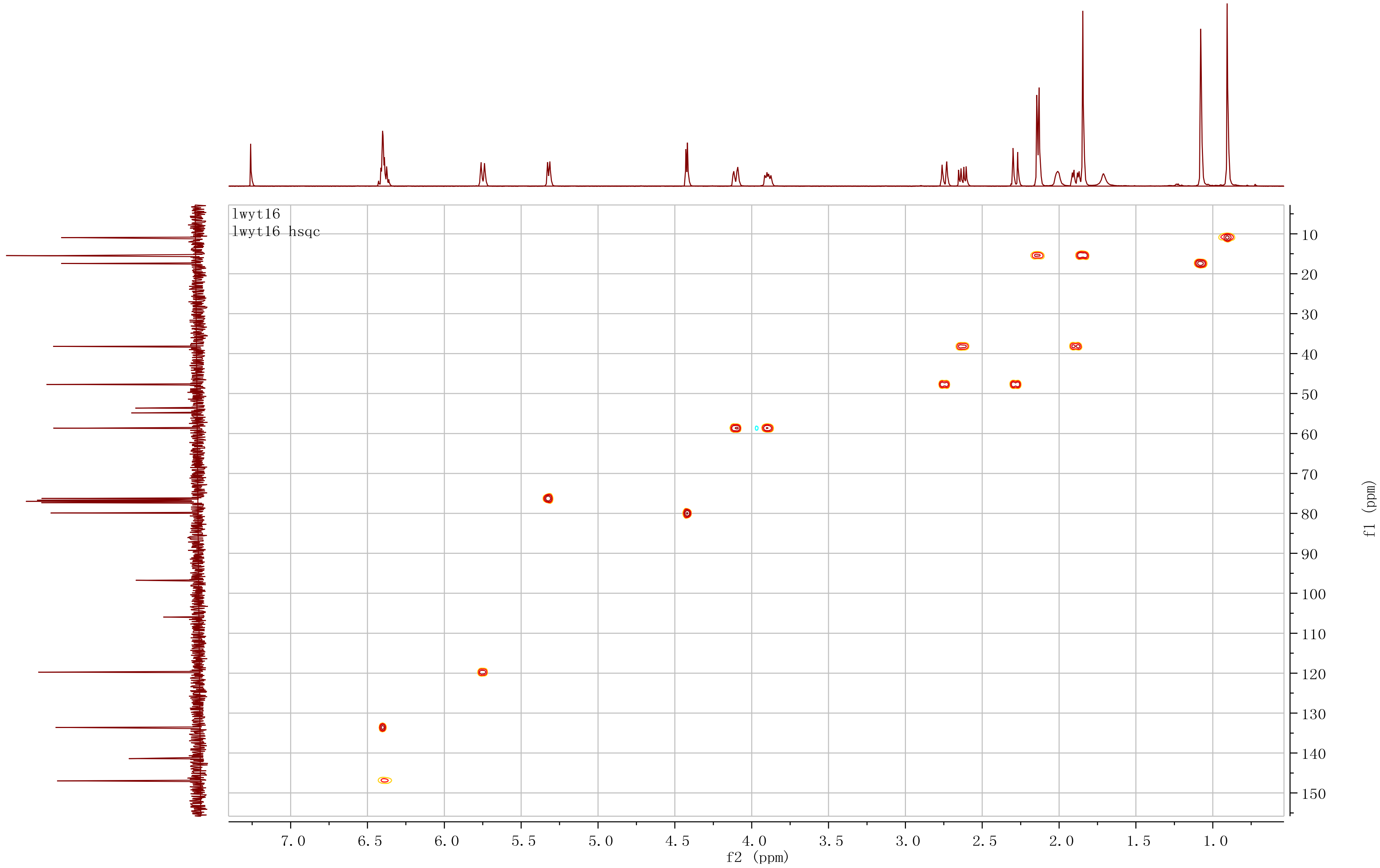

S4. HMBC spectrum (500 MHz, $CDCl_3$) of engleromycone A (**1**)

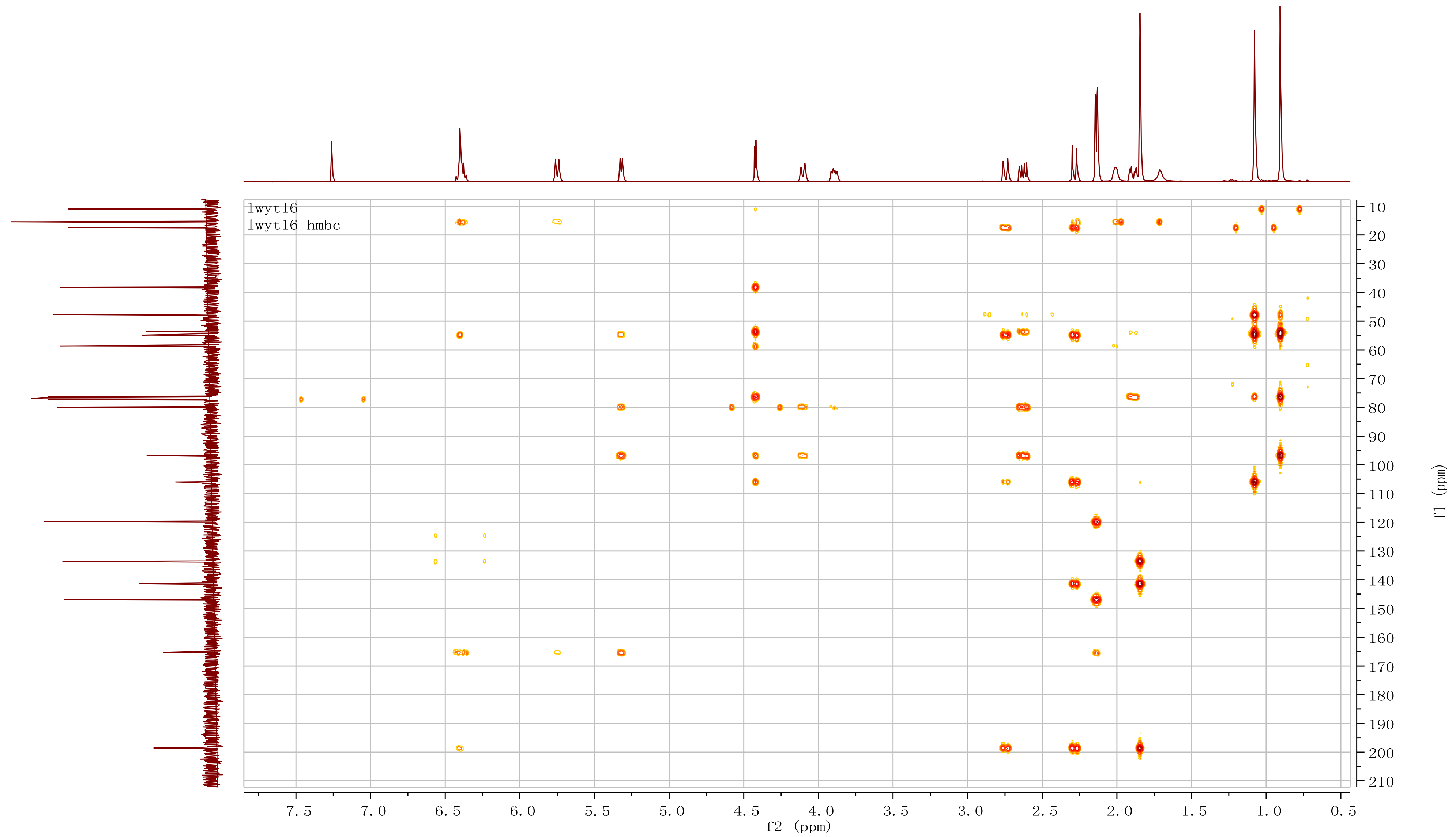

S5. COSY spectrum (500 MHz, $CDCl_3$) of engleromycone A (**1**)

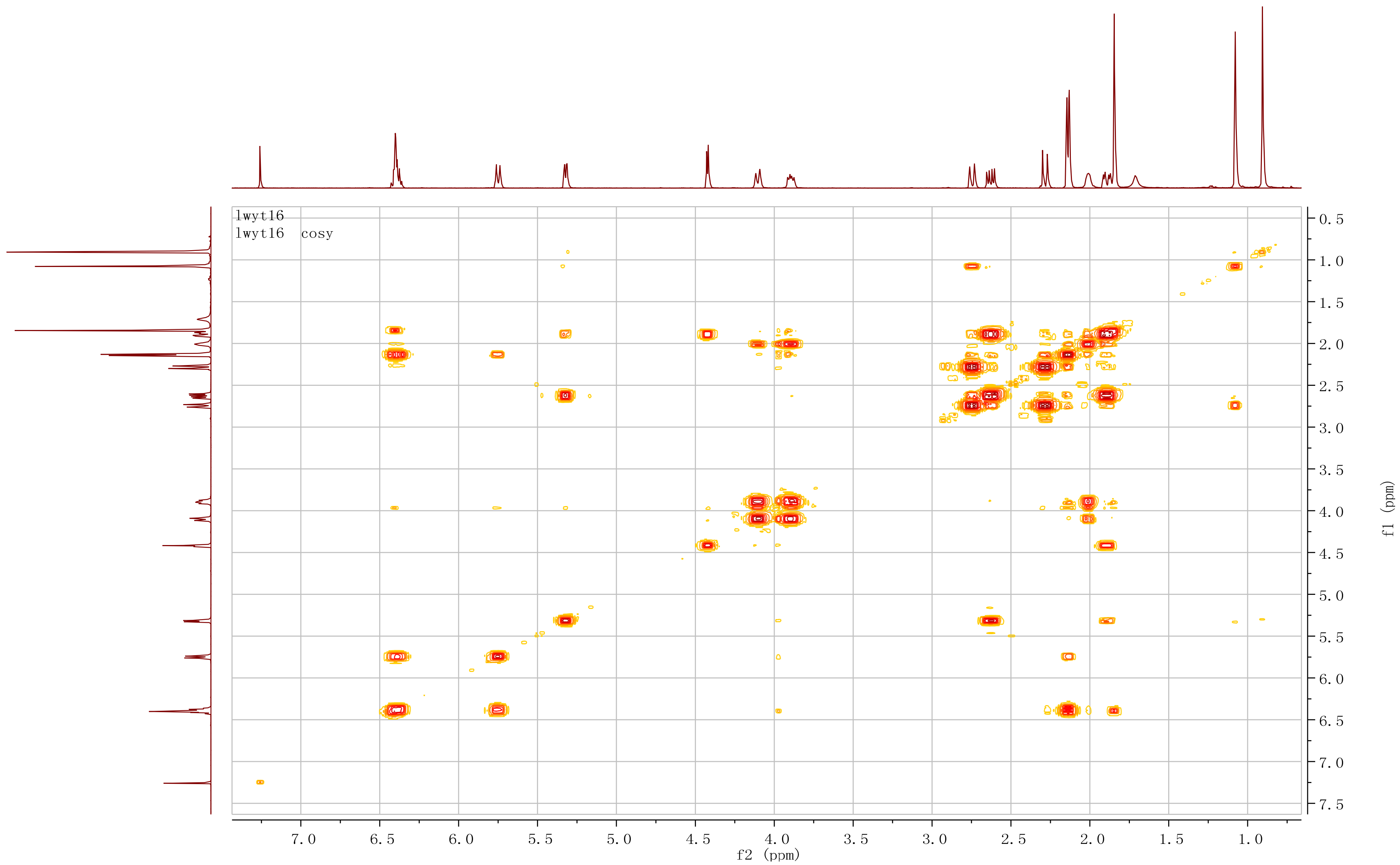

S6. ROSEY spectrum (500 MHz, $CDCl_3$) of engleromycone A (**1**)

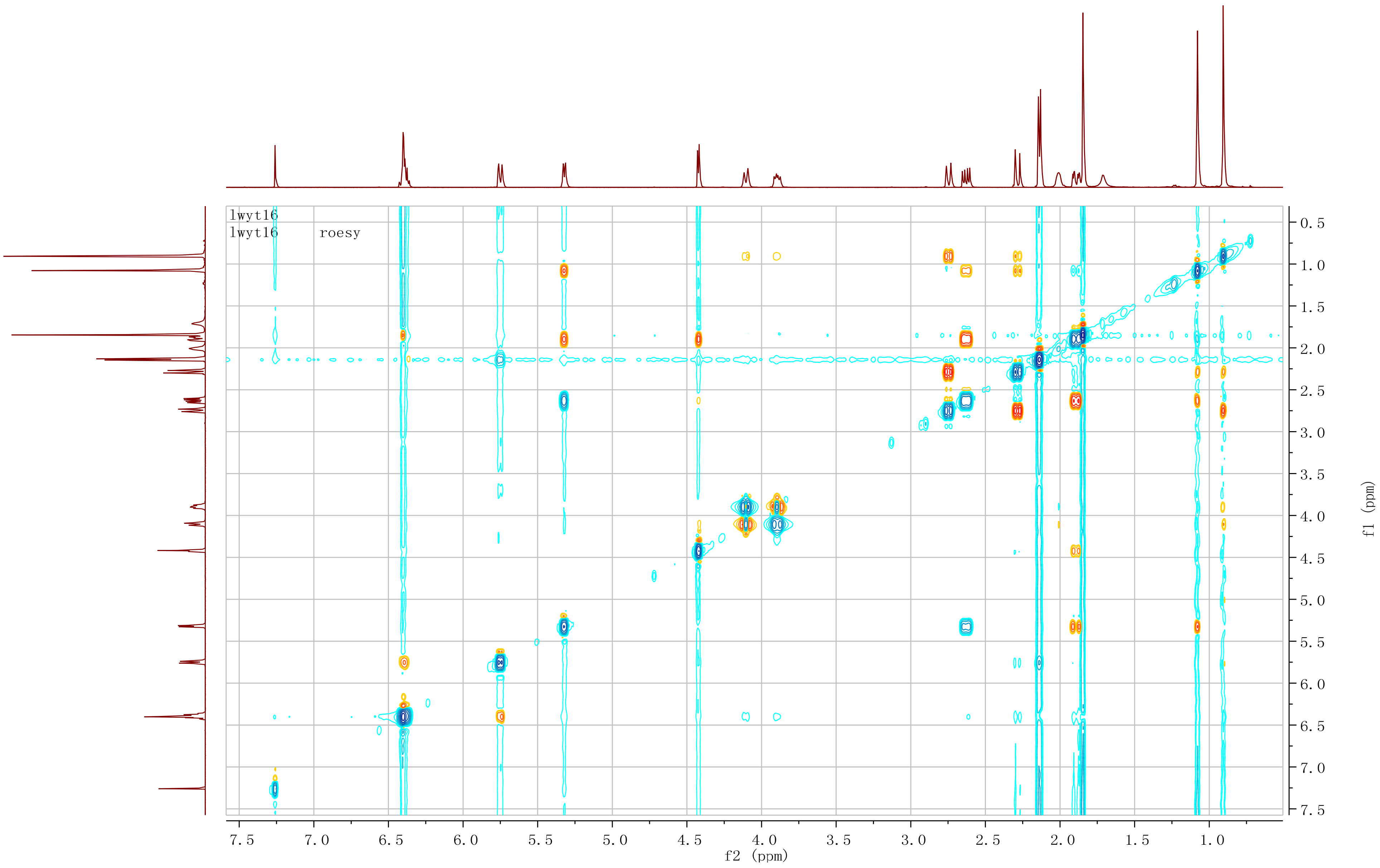

S7. HR-ESI-MS spectrum of engleromycone A (**1**)

Qualitative Analysis Report

**Data Filename**	1403128ESIA19.d	**Sample Name**	lwyt16
**Sample Type**	Sample	**Position**	
**Instrument Name**	Agilent G6230 TOF MS	**User Name**	KIB
**Acq Method**	ESI.m	**Acquired Time**	3/28/2014 3:03:54 PM
**IRM Calibration Status**	Success	**DA Method**	ESIN.m
**Comment**			

Sample Group **Info.**

Acquisition SW 6200 series TOF/6500 series

Version Q-TOF B.05.01 (B5125.1)

User Spectra

Fragmentor Voltage	Collision Energy	Ionization Mode
200	0	ESI

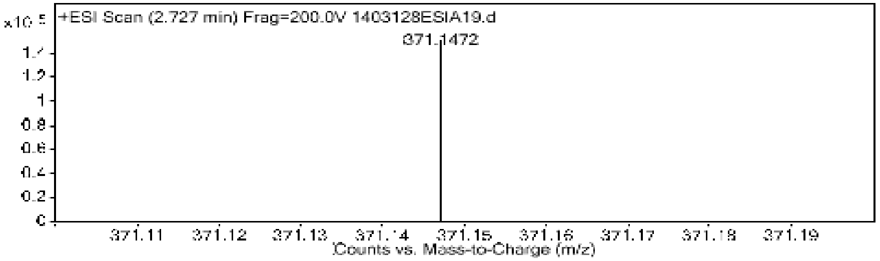

Peak List

m/z	z	Abund
387.1424	1	406345

Formula Calculator Element Limits

Element	Min	Max
C	0	200
H	0	400
O	4	8
Na	1	1

Formula Calculator Results

Formula	CalculatedMass	Mz	Diff.(mDa)	Diff. (ppm)	DBE
C19 H24 Na O6	371.1471	371.1472	-0.1	0.3	7.5

--- End Of Report ---

S8. ^{1}H NMR spectrum (500 MHz, $CDCl_3$) of engleromycone B (**2**)

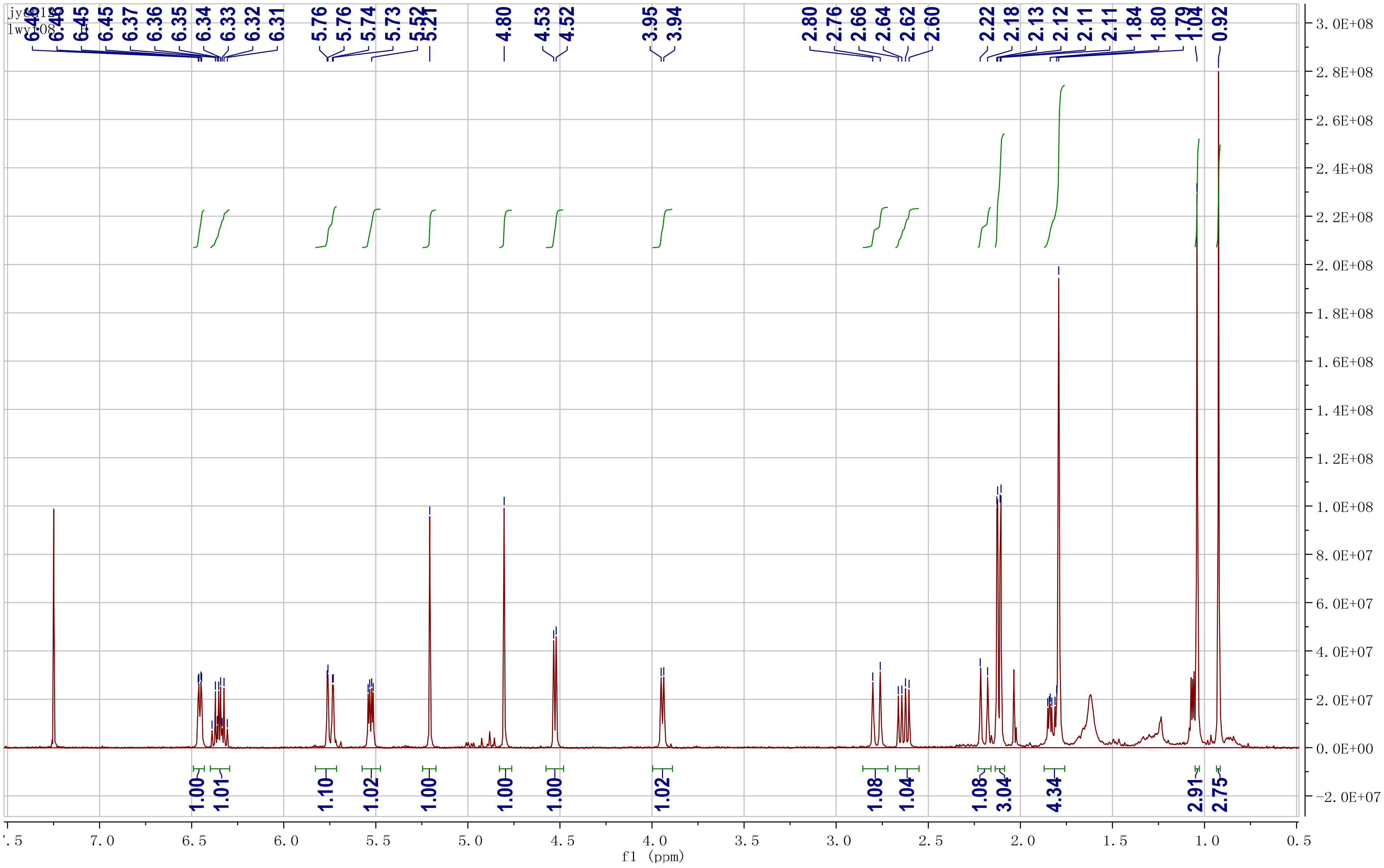

S9. ^{13}C NMR spectrum (100 MHz, $CDCl_3$) of engleromycone B (**2**)

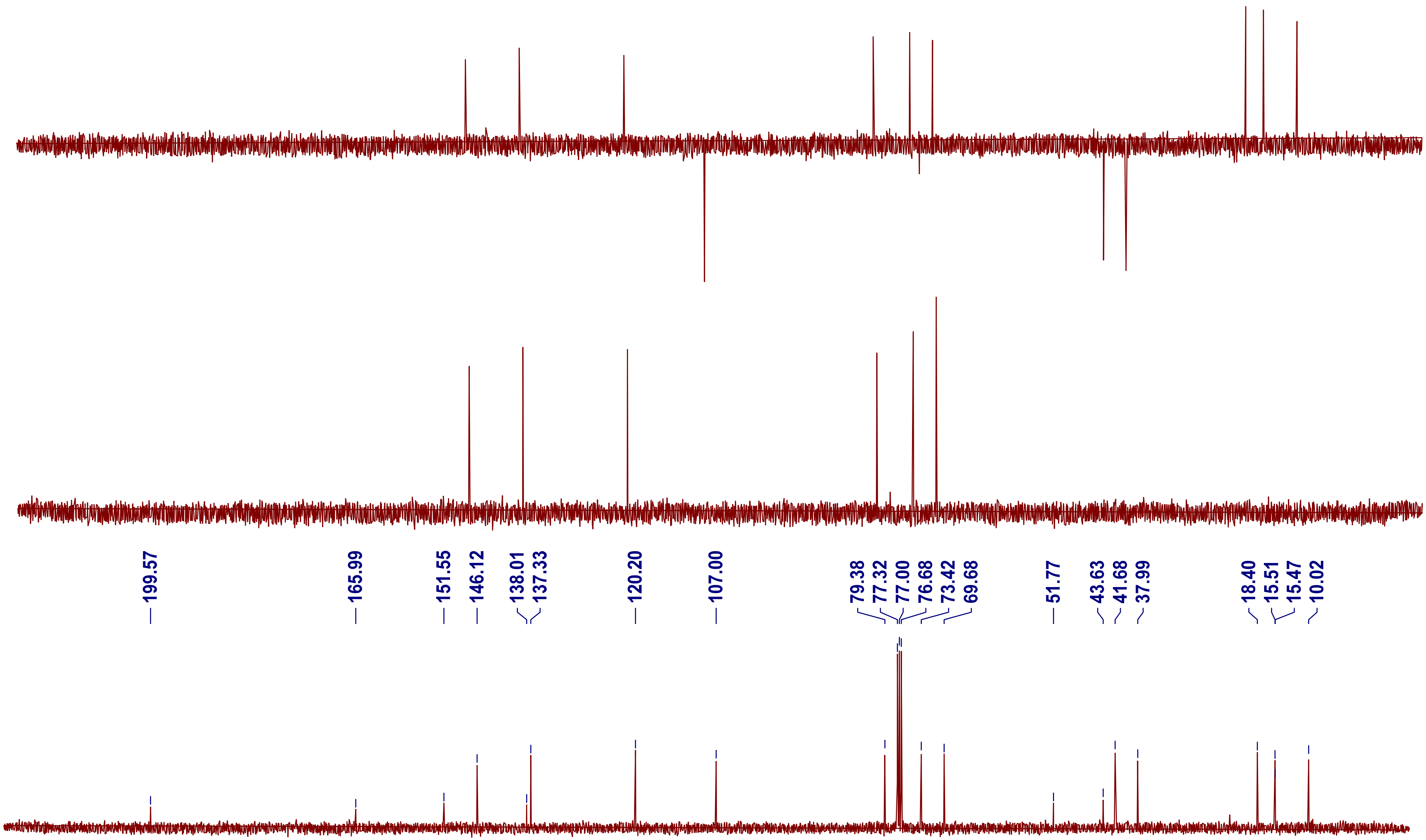

S10. HSQC spectrum (500 MHz, $CDCl_3$) of engleromycone B (**2**)

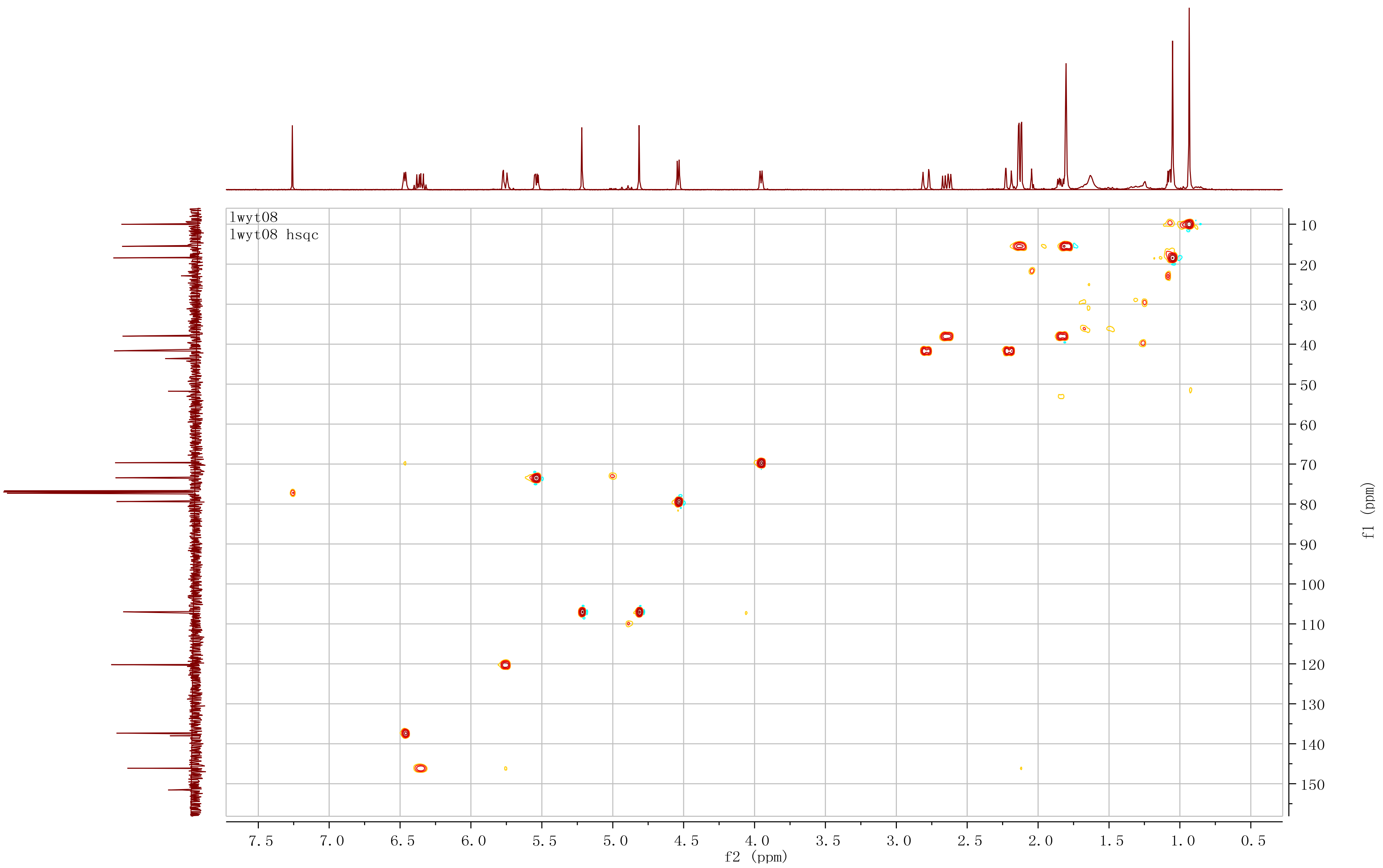

S11. HMBC spectrum (500 MHz, $CDCl_3$) of engleromycone B (**2**)

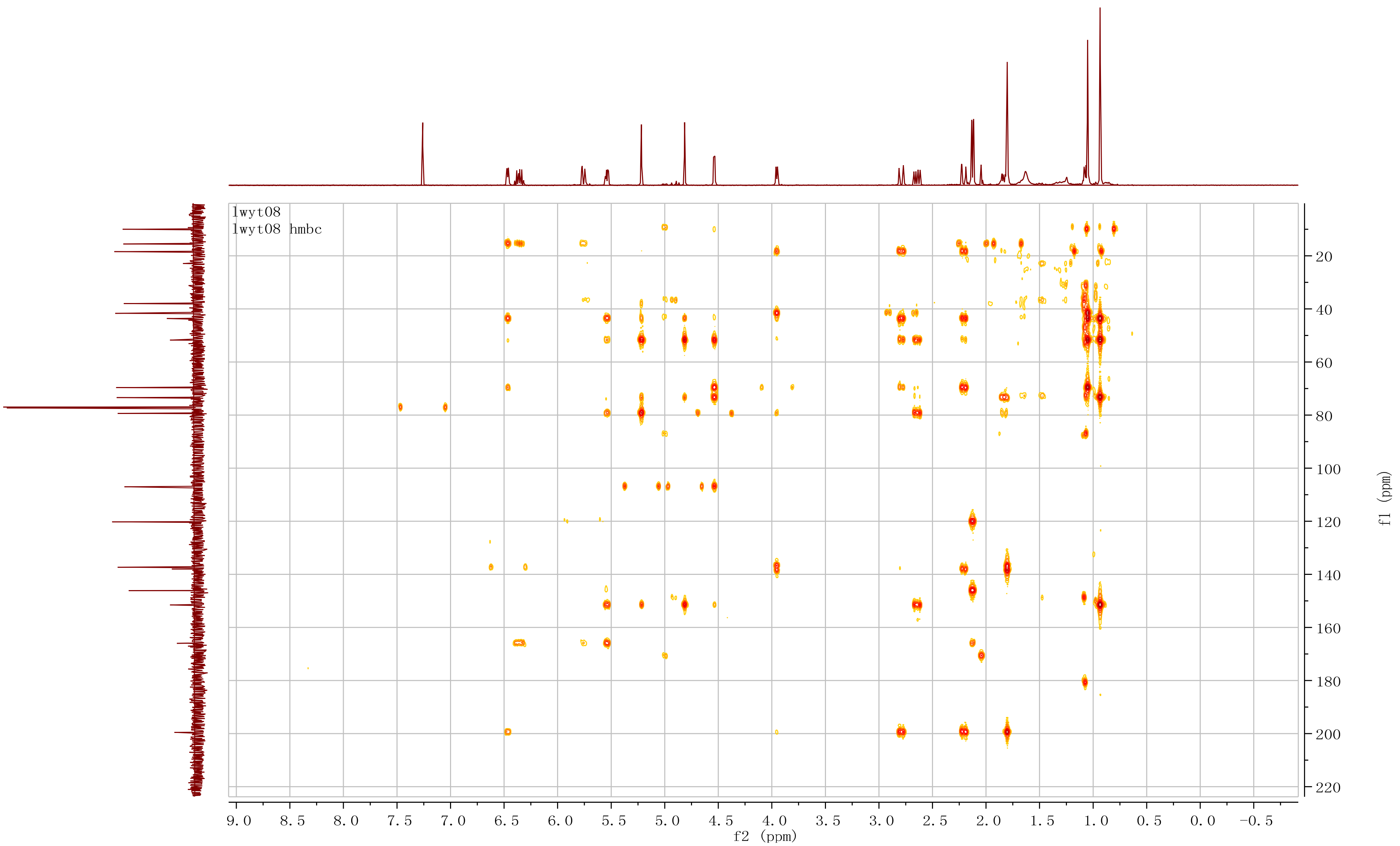

S12. COSY spectrum (500 MHz, $CDCl_3$) of engleromycone B (**2**)

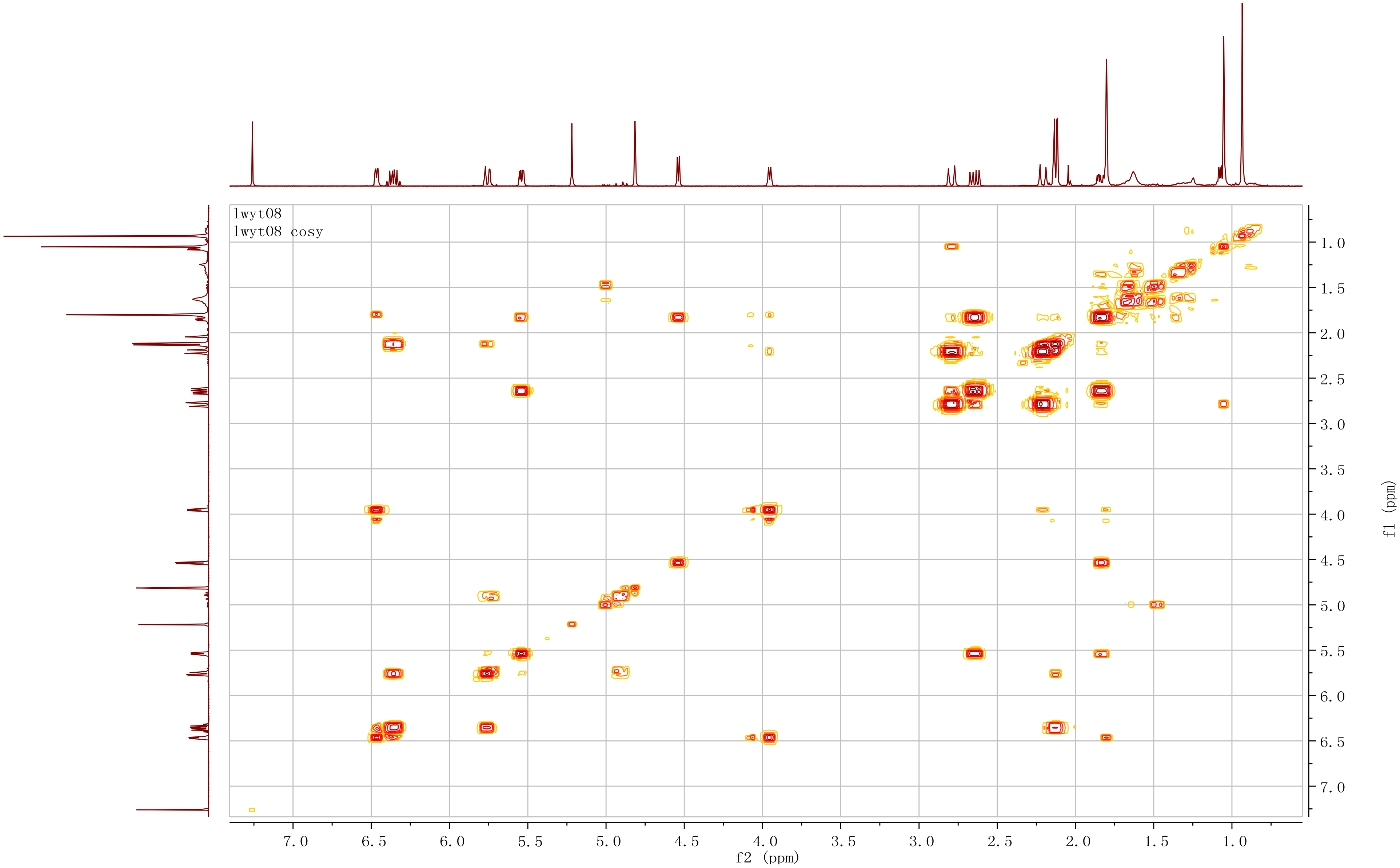

S13. ROSEY spectrum (500 MHz, $CDCl_3$) of engleromycone B (**2**)

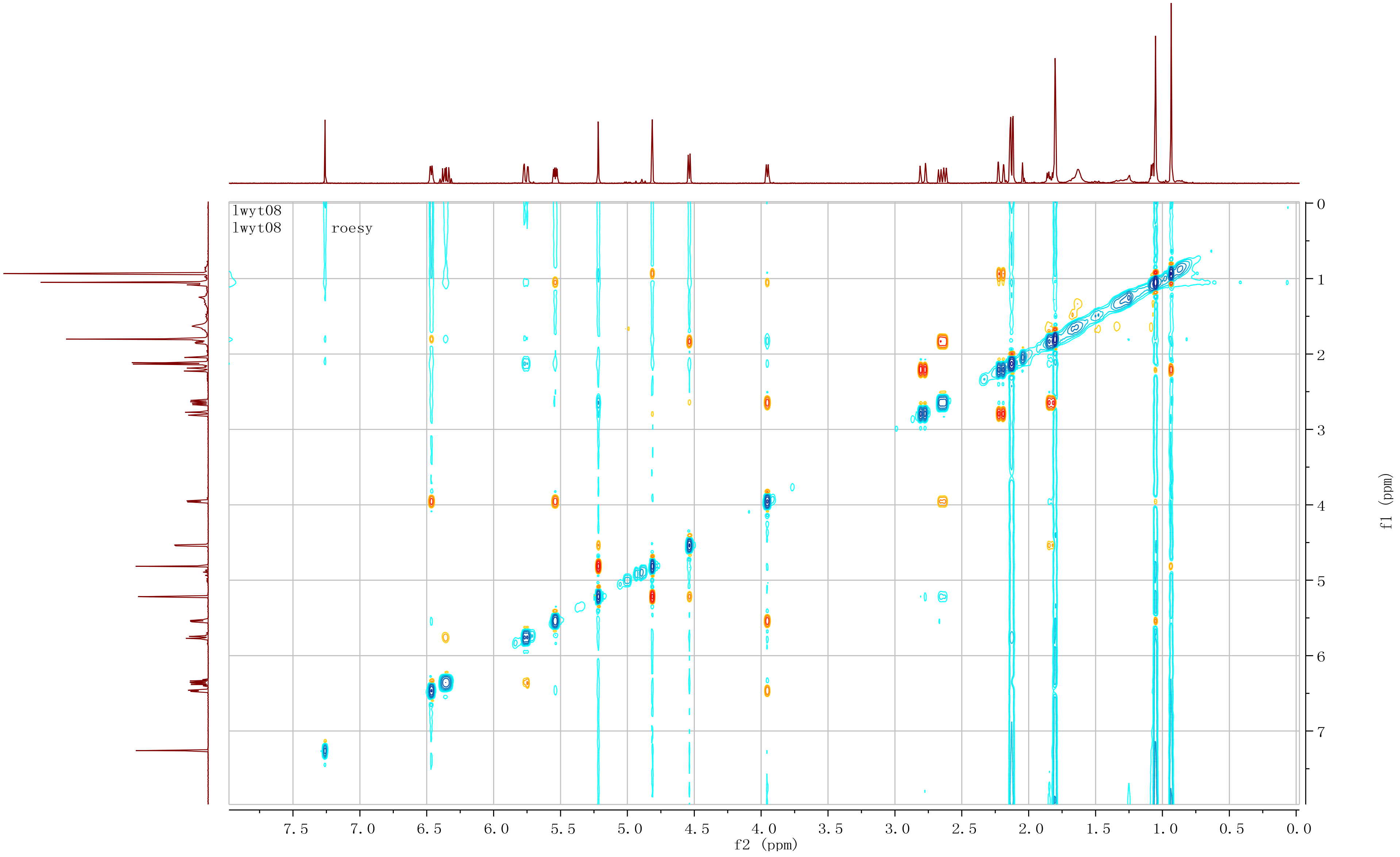

S14. HR-ESI-MS spectrum of engleromycone B (**2**)

Qualitative Analysis Report

**Data Filename**	140307ESIA1.d	**Sample Name**	lwyt08
**Sample Type**	Sample	**Position**	
**Instrument Name**	Agilent G6230 TOF MS	**User Name**	KIB
**Acq Method**	ESI.m	**Acquired Time**	3/6/2014 10:25:15 AM
**IRM Calibration Status**	Success	**DA Method**	ESIN.m
**Comment**			

Sample Group **Info.**
Acquisition SW 6200 series TOF/6500 series
Version Q-TOF B.05.01 (B5125.1)

User Spectra

Fragmentor Voltage	Collision Energy	Ionization Mode
200	0	ESI

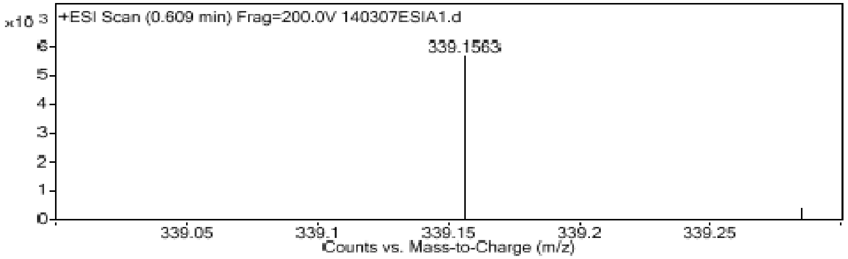

Peak List

m/z	z	Abund
274.2742	1	287953.44

Formula Calculator Element Limits

Element	Min	Max
C	0	200
H	0	400
O	3	5
Na	1	1

Formula Calculator Results

Formula	CalculatedMass	Mz	Diff.(mDa)	Diff. (ppm)	DBE
C19 H24 Na O4	339.1572	339.1563	0.9	2.7	7.5

--- End Of Report ---

S15. ^{1}H NMR spectrum (600 MHz,acetone-d_6) of infuscol F (**11**)

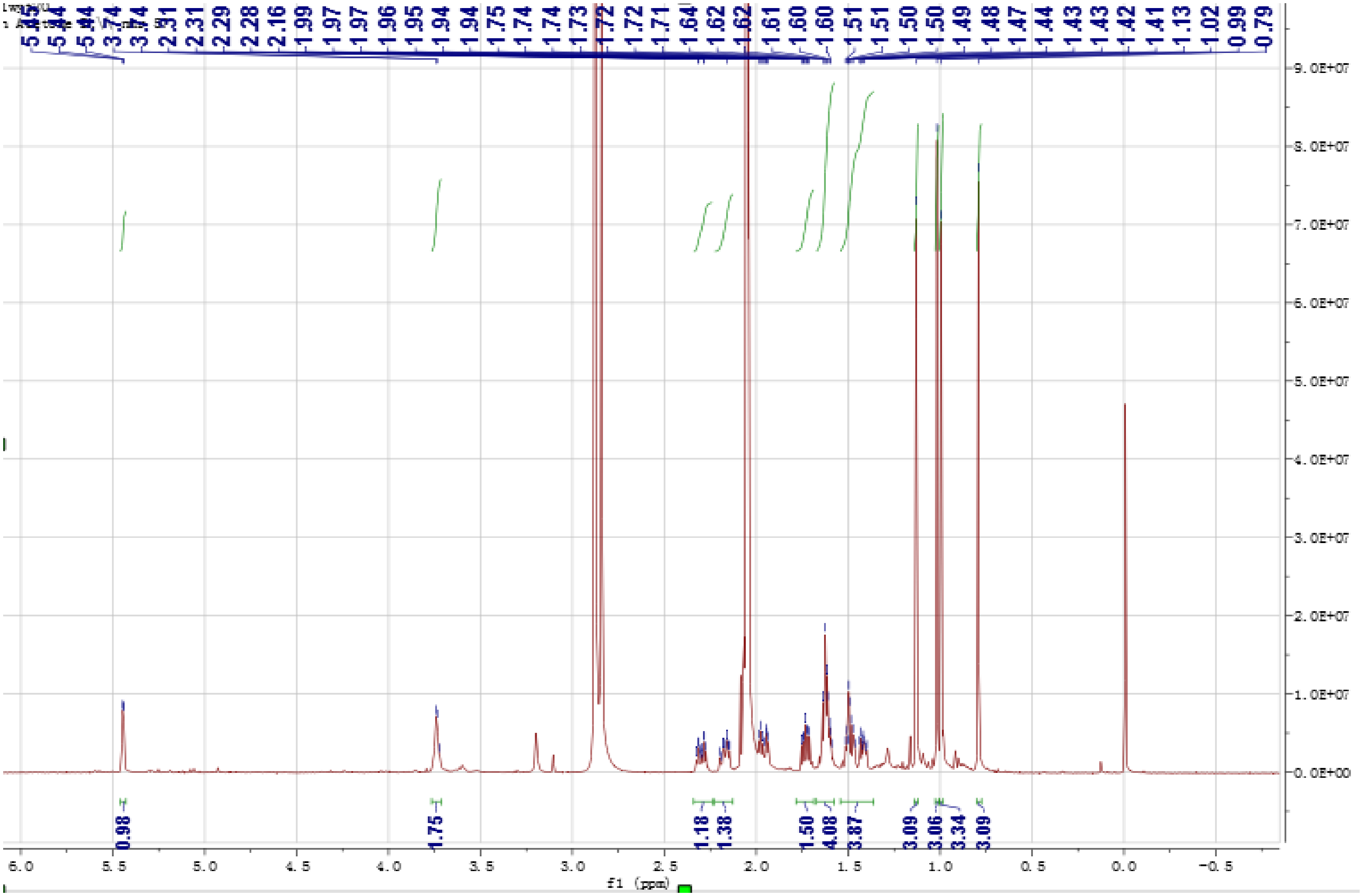

S16. ^{13}C NMR spectrum (150 MHz, acetone-d$_6$) of infuscol F (**11**)

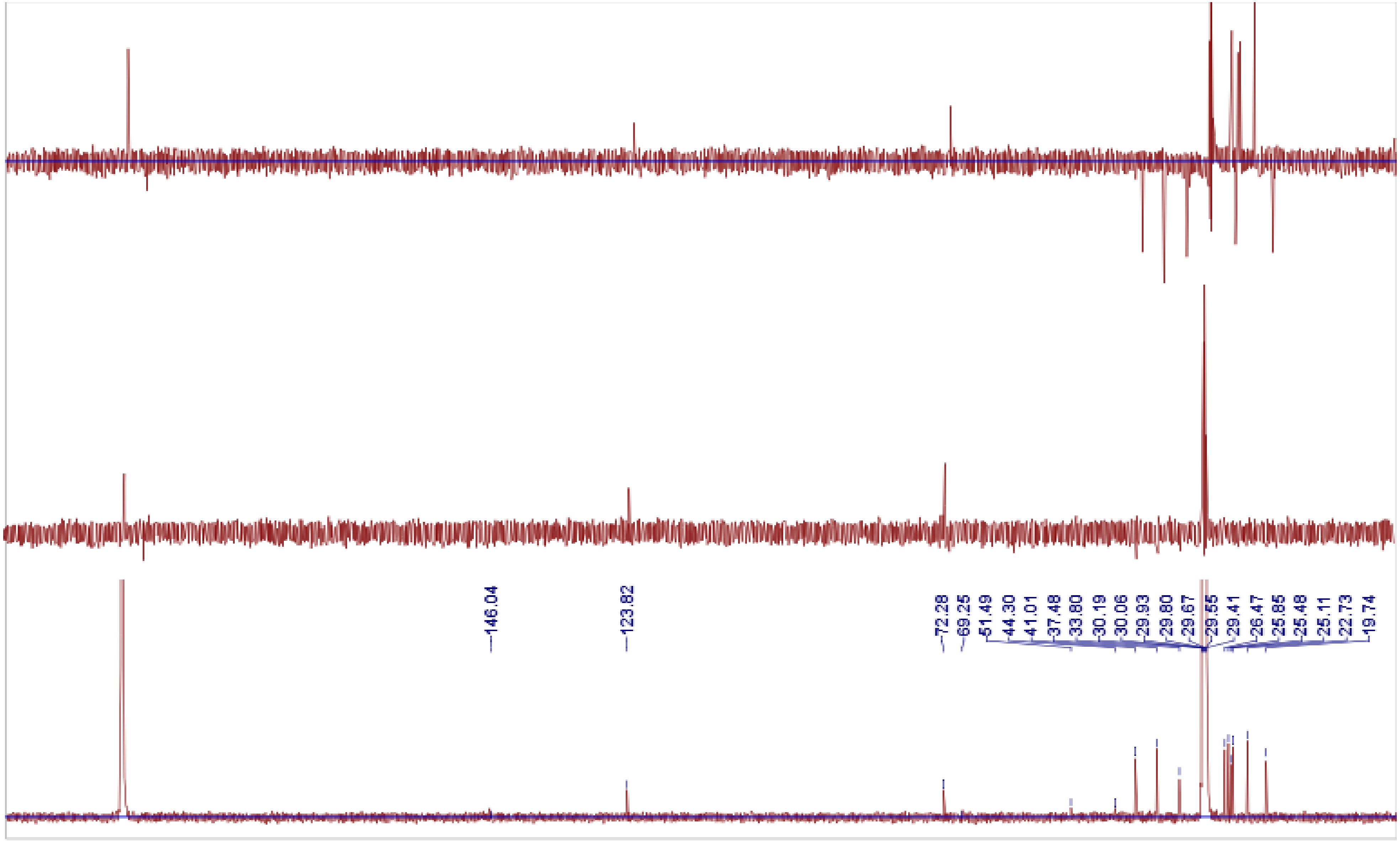

S17. HSQC spectrum (600 MHz,acetone-d$_6$) of infuscol F (**11**)

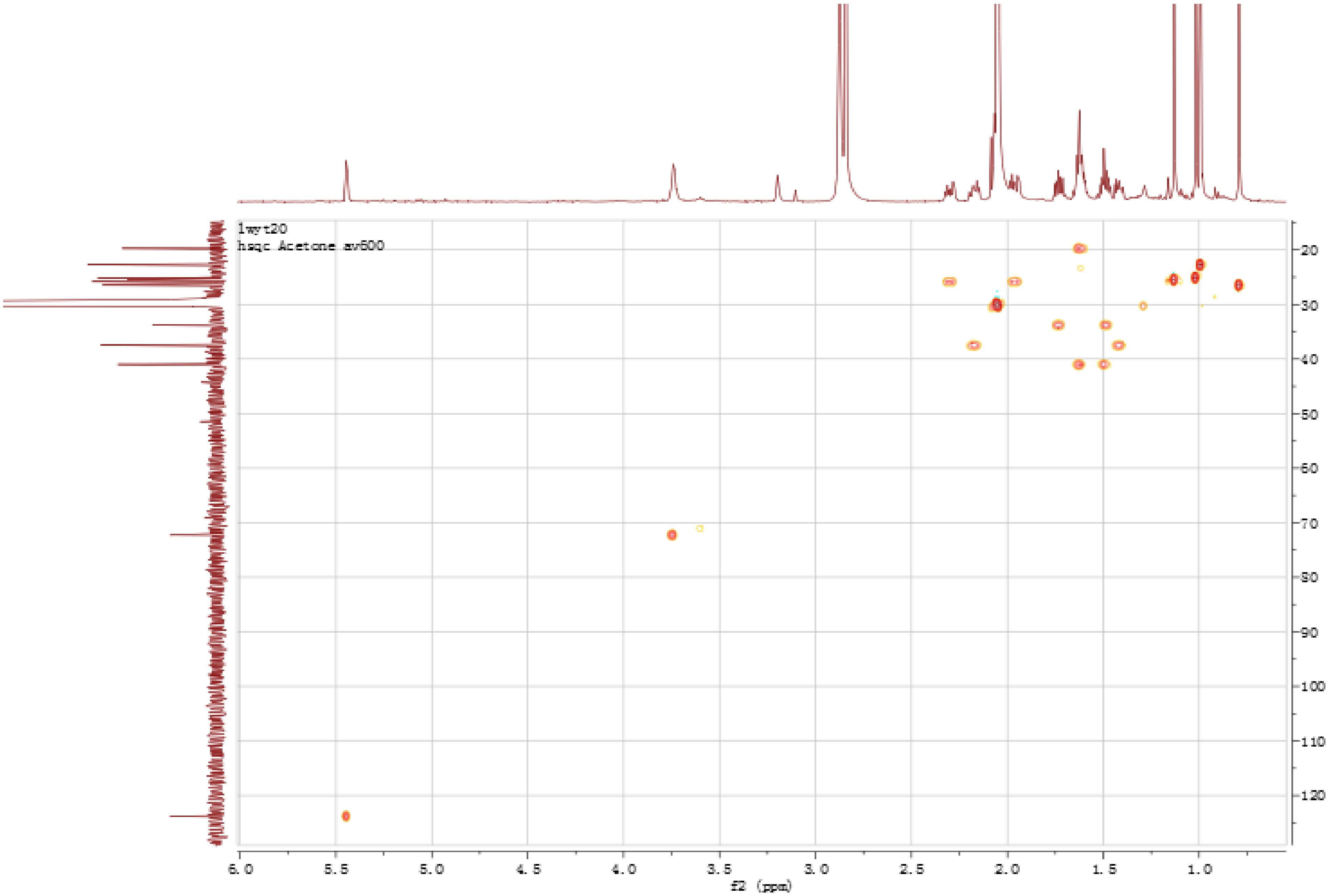

S18. HMBC spectrum (600 MHz,acetone-d_6) of infuscol F (**11**)

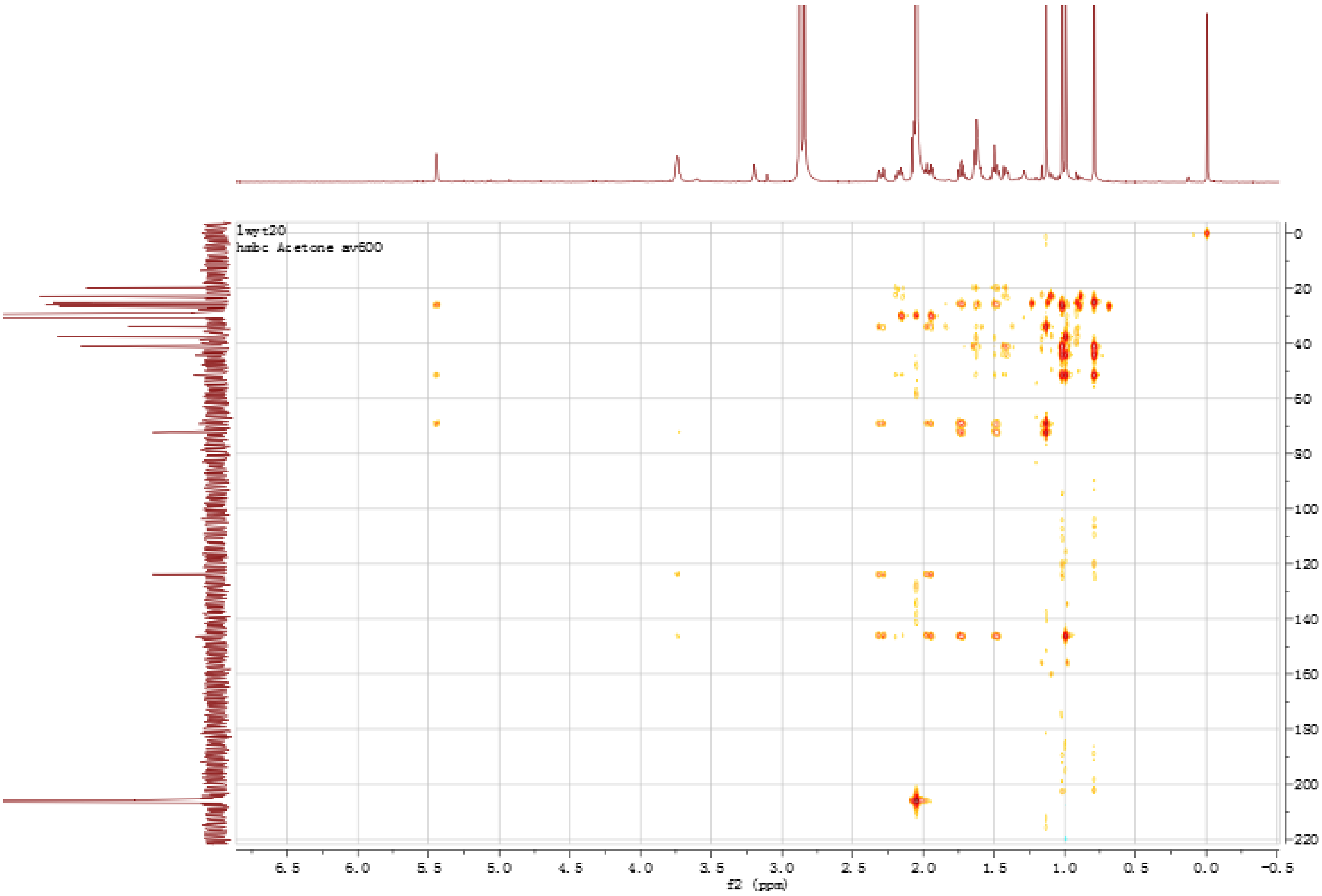

S19. COSY spectrum (600 MHz,acetone-d_6) of infuscol F (**11**)

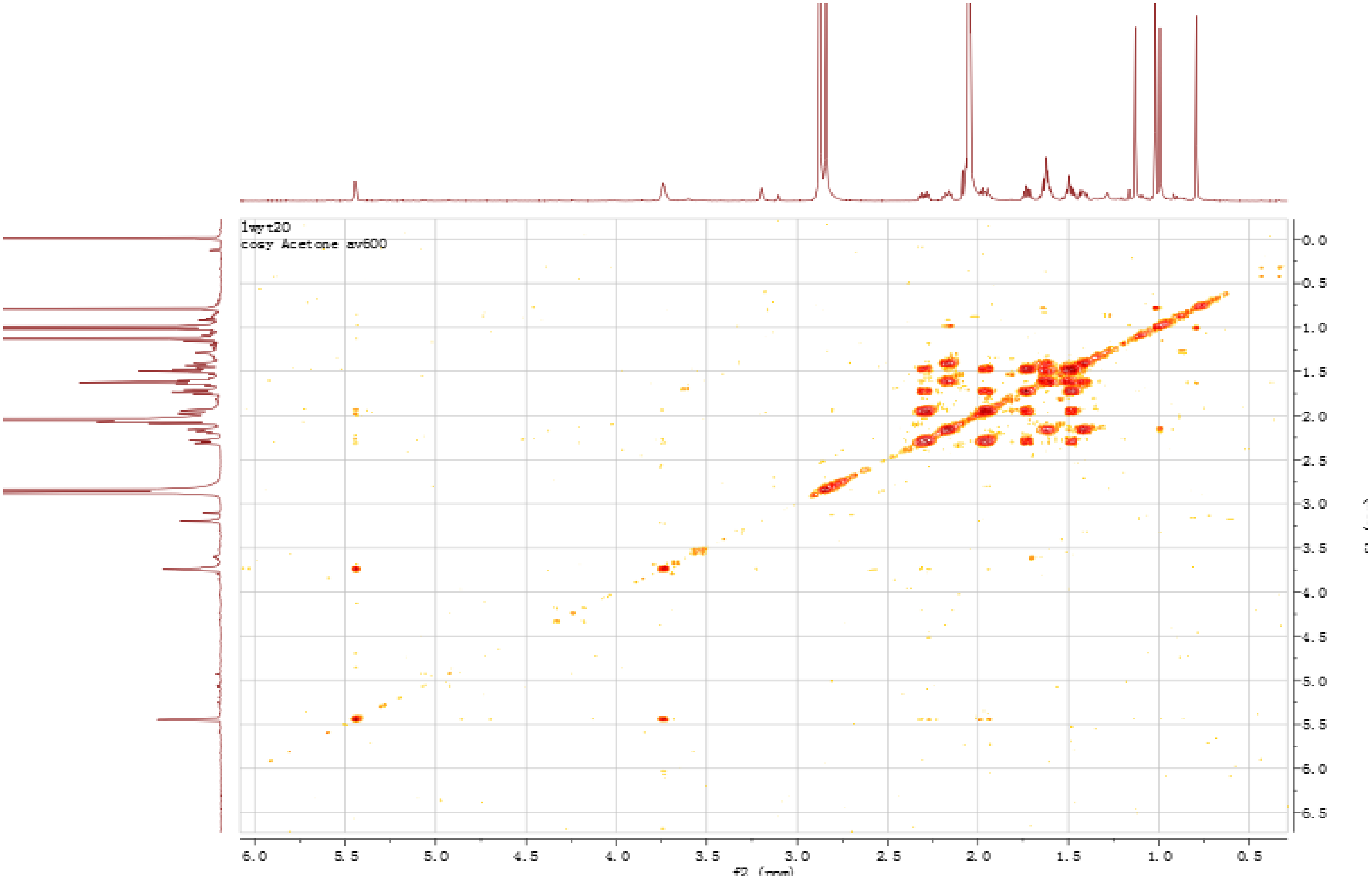

S20. ROSEY spectrum (600 MHz,acetone-d$_6$) of infuscol F (**11**)

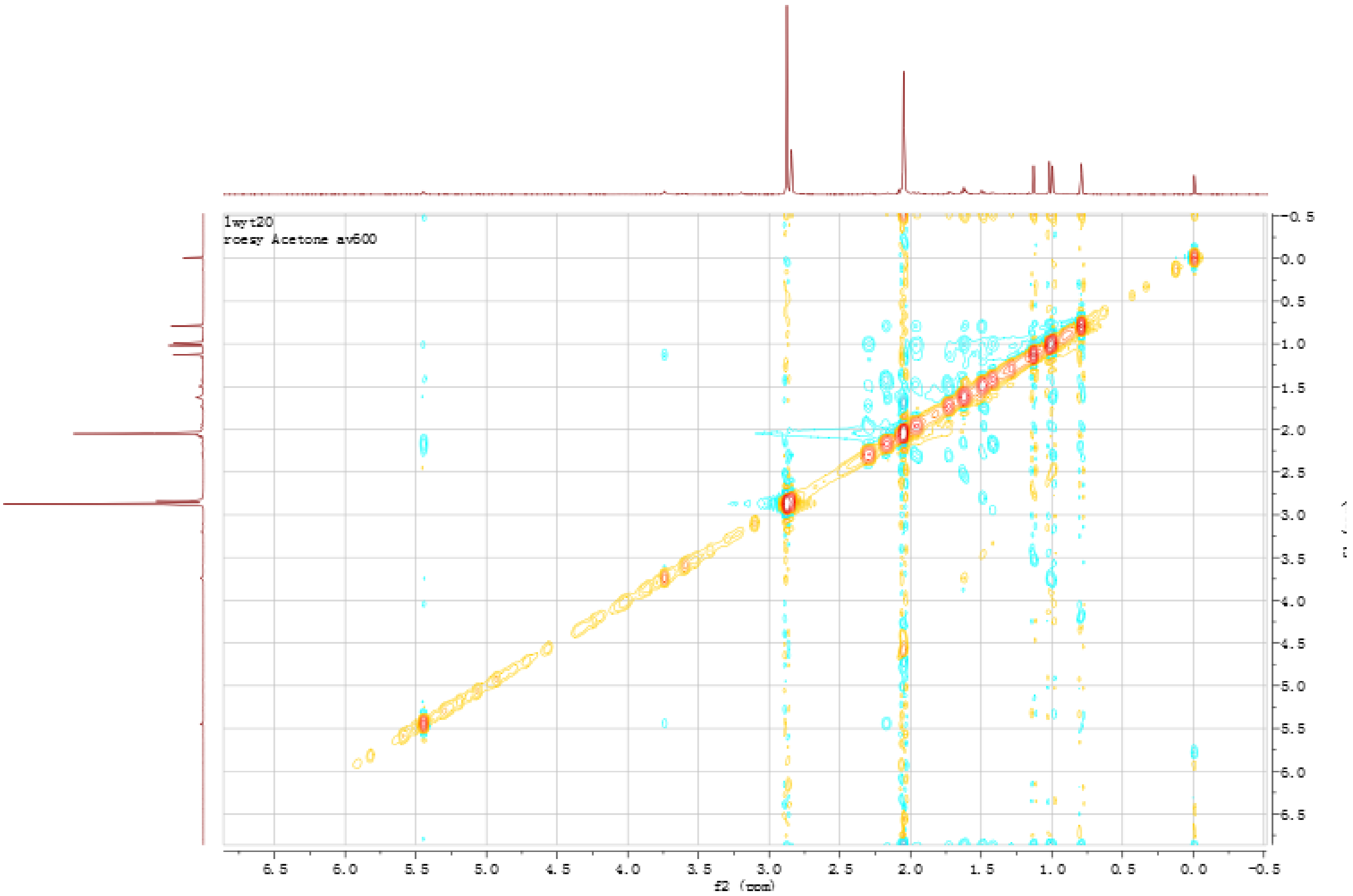

S21. HR-ESI-MS spectrum of infuscol F (**11**)

Qualitative Analysis Report

**Data Filename**	140307ESIA2.d	**Sample Name**	lwyt20
**Sample Type**	Sample	**Position**	
**Instrument Name**	Agilent G6230 TOF MS	**User Name**	KIB
**Acq Method**	ESI.m	**Acquired Time**	3/6/2014 10:37:58 AM
**IRM Calibration Status**	Success	**DA Method**	ESIN.m
**Comment**			

Sample Group **Info.**
Acquisition SW 6200 series TOF/6500 series
Version Q-TOF B.05.01 (B5125.1)

User Spectra

Fragmentor Voltage	Collision Energy	Ionization Mode
200	0	ESI

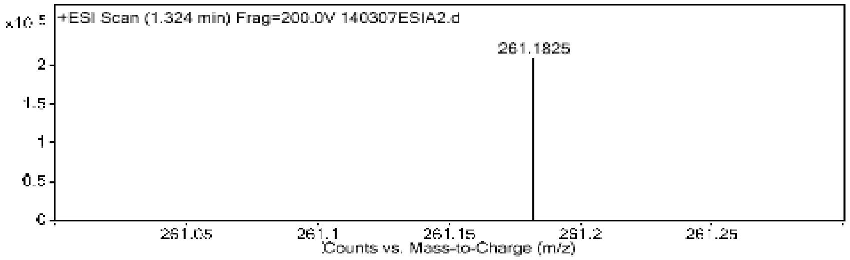

Peak List

m/z	z	Abund	Formula	Ion
261.1825	1	208464.28	C15 H26 Na O2	M+

Formula Calculator Element Limits

Element	Min	Max
C	0	200
H	0	400
O	1	3
Na	1	1

Formula Calculator Results

Formula	CalculatedMass	Mz	Diff.(mDa)	Diff. (ppm)	DBE
C15 H26 Na O2	261.1830	261.1825	0.5	1.9	2.5

--- End Of Report ---
